# The *Toxoplasma gondii* mitochondrial transporter ABCB7L is essential for the biogenesis of cytosolic and nuclear iron-sulfur cluster proteins and cytosolic translation

**DOI:** 10.1128/mbio.00872-24

**Published:** 2024-08-29

**Authors:** Andrew E. Maclean, Megan A. Sloan, Eléa A. Renaud, Blythe E. Argyle, William H. Lewis, Jana Ovciarikova, Vincent Demolombe, Ross F. Waller, Sébastien Besteiro, Lilach Sheiner

**Affiliations:** 1 Wellcome Centre for Integrative Parasitology, University of Glasgow, Glasgow, United Kingdom; 2 School of Infection and Immunity, University of Glasgow, Glasgow, United Kingdom; 3 LPHI, Univ Montpellier, CNRS, INSERM, Montpellier, France; 4 Department of Biochemistry, University of Cambridge, Cambridge, United Kingdom; 5 IPSiM, Univ Montpellier, CNRS, INRAE, Institut Agro, Montpellier, France; Albert Einstein College of Medicine, Bronx, New York, USA

**Keywords:** *Toxoplasma gondii*, iron-sulfur cluster, ABCB7L, cytosolic iron-sulfur assembly pathway, cofactor biosynthesis, mitochondria

## Abstract

Iron-sulfur (Fe-S) clusters are ubiquitous inorganic cofactors required for numerous essential cellular pathways. Since they cannot be scavenged from the environment, Fe-S clusters are synthesized *de novo* in cellular compartments such as the apicoplast, mitochondrion, and cytosol. The cytosolic Fe-S cluster biosynthesis pathway relies on the transport of an intermediate from the mitochondrial pathway. An ATP-binding cassette (ABC) transporter called ABCB7 is responsible for this role in numerous commonly studied organisms, but its role in the medically important apicomplexan parasites has not yet been studied. Here we identify and characterize a *Toxoplasma gondii* ABCB7 homolog, which we name ABCB7-like (ABCB7L). Genetic depletion shows that it is essential for parasite growth and that its disruption triggers partial stage conversion. Characterization of the knock-down line highlights a defect in the biogenesis of cytosolic and nuclear Fe-S proteins leading to defects in protein translation and other pathways including DNA and RNA replication and metabolism. Our work provides support for a broad conservation of the connection between mitochondrial and cytosolic pathways in Fe-S cluster biosynthesis and reveals its importance for parasite survival.

**IMPORTANCE:**

Iron-sulfur (Fe-S) clusters are inorganic cofactors of proteins that play key roles in numerous essential biological processes, for example, respiration and DNA replication. Cells possess dedicated biosynthetic pathways to assemble Fe-S clusters, including a pathway in the mitochondrion and cytosol. A single transporter, called ABCB7, connects these two pathways, allowing an essential intermediate generated by the mitochondrial pathway to be used in the cytosolic pathway. Cytosolic and nuclear Fe-S proteins are dependent on the mitochondrial pathway, mediated by ABCB7, in numerous organisms studied to date. Here, we study the role of a homolog of ABCB7, which we name ABCB7-like (ABCB7L), in the ubiquitous unicellular apicomplexan parasite *Toxoplasma gondii*. We generated a depletion mutant of *Toxoplasma* ABCB7L and showed its importance for parasite fitness. Using comparative quantitative proteomic analysis and experimental validation of the mutants, we show that ABCB7L is required for cytosolic and nuclear, but not mitochondrial, Fe-S protein biogenesis. Our study supports the conservation of a protein homologous to ABCB7 and which has a similar function in apicomplexan parasites and provides insight into an understudied aspect of parasite metabolism.

## INTRODUCTION

Iron-sulfur (Fe-S) clusters are ubiquitous inorganic metallocofactors, made up of geometric arrangements of iron and sulfur atoms, that are essential across cellular life. Due to their electron transfer capabilities, they are important components of enzymes required for critical cellular functions (1). Many mitochondrial Fe-S proteins are components of the mitochondrial electron transport chain (mETC) and are involved in respiration. Cytosolic and nuclear Fe-S proteins include enzymes that play a role in DNA replication and repair, amino acid biosynthesis, tRNA modification, and protein translation (2). Therefore, it is essential for cellular life that Fe-S clusters are synthesized and trafficked to their correct cellular location.

**Fig 1:**
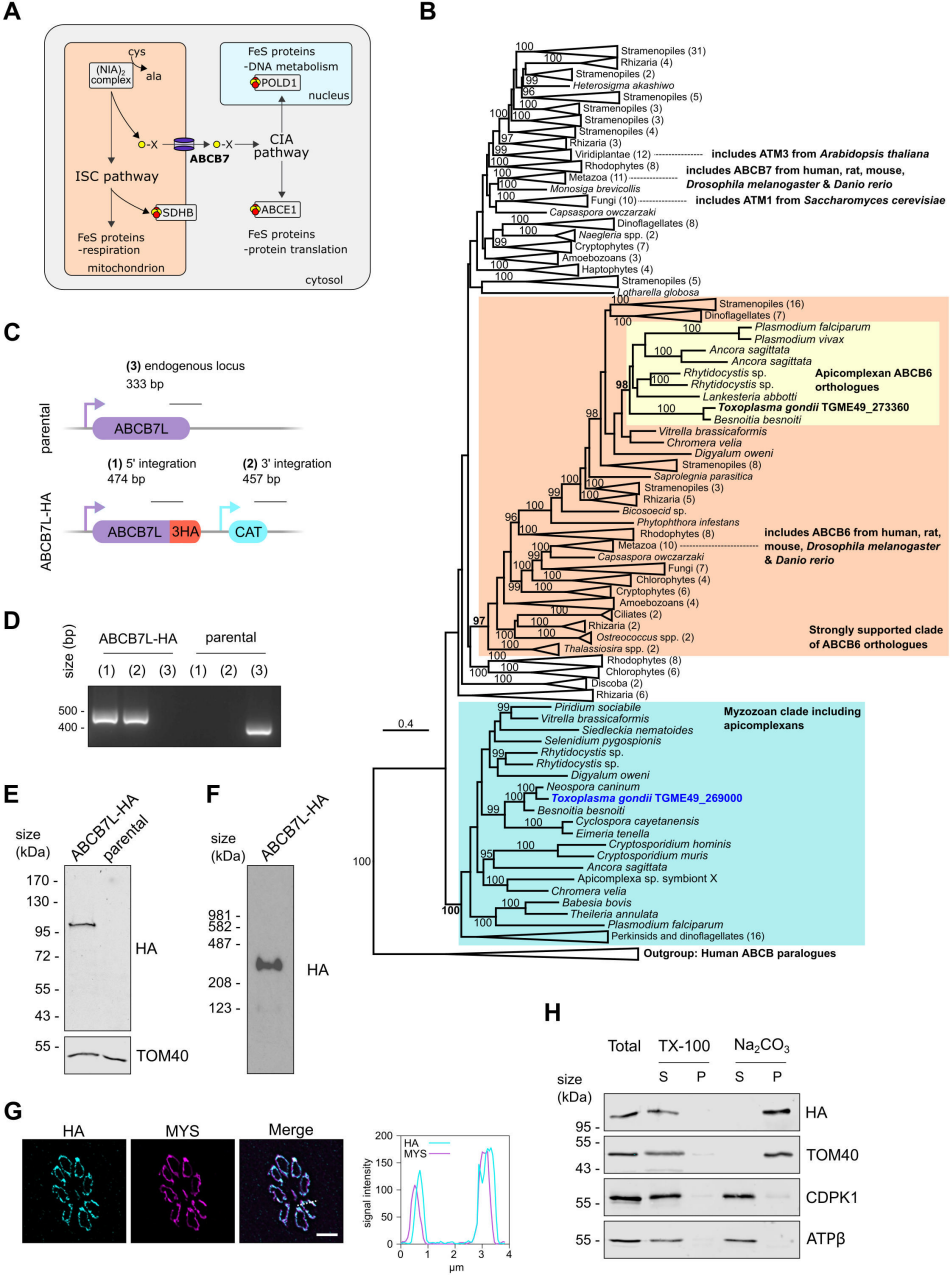
*Toxoplasma* ABCB7L is an integral membrane protein that localizes to the mitochondrion. (**A**) Schematic of the mitochondrial ISC and cytosolic CIA Fe-S assembly pathways. ABCB7 (bold) connects these two pathways. Fe-S-containing proteins evaluated in this study are shown (POLD1, ABCE1, and SDHB). (**B**) A phylogeny inferred from amino acid sequences of ABCB homologs from various eukaryotes closely related to characterized ABCB6 and ABCB7 homologs from selected model organisms (positions in tree highlighted by dashed lines). The tree is rooted in ABCB paralogs of ABCB6 and ABCB7 from humans. Support values, displayed as percentages, were generated from 1,000 ultrafast bootstrap replicates (8), and only support values ≥95, indicating a strongly supported clade are shown. A strongly supported clade (support value = 100) of myzozoa (including apicomplexans) sequences is highlighted (blue) that includes TGGT1_269000 from *Toxoplasma gondii*. A strongly supported clade (support value = 98) of apicomplexan sequences, including TGGT1_273360 from *Toxoplasma gondii,* is also highlighted (yellow), which forms part of a larger strongly supported clade (support value = 97) with ABCB6 from selected model organisms (highlighted in red) and therefore all sequences within this clade are inferred to be ABCB6 orthologs. The scale bar represents the number of amino acid substitutions per site. A fully expanded version of this tree is provided in Fig. S2. (**C**) Schematic of the strategy is used to C-terminally HA-epitope tag the *Tg*ABCB7L protein. The expected size of integration PCRs is shown. (**D**) PCR to test the integration of HA-epitope tag and CAT selection cassette into the endogenous locus, as outlined in panel **C**. (**E**) Immunoblot analysis of whole-cell lysate extracted from ABCB7-HA and parental parasites. Samples were separated by SDS-PAGE, blotted, and detected using anti-HA, to visualize ABCB7L-HA, and anti-TOM40 as a loading control. (**F**) BN-PAGE analysis of ABCB7L-HA parasites extracted in 1% βDDM, immunolabeled with anti-HA. (**G**) Immunofluorescence assay analysis of ABCB7-HA parasites, labeled with anti-HA to detect ABCB7L-HA (cyan), showing co-localization with the mitochondrial marker protein MYS (magenta). The scale bar is 5 µm. The graph shows the signal intensity for both channels along the dotted line depicted in the merged image. (**H**) Immunoblot analysis of ABCB7L-HA parasites treated with 1% Triton X-100 (TX-100) or sodium carbonate (Na_2_CO_3_) at pH 11.5 and separated by centrifugation to give a supernatant (S) and pellet (P) fraction. A total fraction, before treatment, is also analyzed. ABCB7L-HA is found in the pellet fraction of Na_2_CO_3_, like the integral outer membrane protein TOM40. The peripheral mitochondrial matrix subunit of ATP synthase, ATPβ, and the cytosolic protein CDPK1 are found in the soluble supernatant fraction.

Fe-S clusters cannot be scavenged from the environment, nor a host cell in the case of intracellular parasites. Therefore, all cells contain dedicated biosynthetic pathways. These directly involve over 30 protein components localized to numerous cell compartments. These proteins are required to bring together and assemble sulfide ions (S^2−^) and ferrous (Fe^2+^) or ferric (Fe^3+^) iron into nascent Fe-S clusters and then deliver them to numerous client proteins (3). Organisms that contain an endosymbiotic plastid typically contain three such biosynthetic pathways: the mitochondrial iron-sulfur cluster assembly pathway (the ISC pathway), the plastid sulfur mobilization pathway (the SUF pathway), and the cytosolic iron-sulfur assembly pathway (the CIA pathway). While the SUF pathway provides Fe-S clusters exclusively for a handful of plastid enzymes, the mitochondrial ISC and cytosolic CIA pathway are connected (Fig. 1A). A mitochondrial cysteine desulfurase (NFS1) interacts with stabilizing protein ISD11 and the mitochondrial acyl carrier protein ACP (NFS1-ISD11-ACP, or NIA) to form a dimeric complex called (NIA)_2_ (4–6). This complex liberates sulfur from cysteine, which is then combined with iron and assembled into Fe-S clusters on the mitochondrial scaffold protein ISU1, in a process involving ferredoxin and frataxin. A number of extra “carrier” proteins then deliver and insert the nascent cluster into mitochondrial Fe-S proteins. The CIA pathway is also dependent on mitochondrial NFS1 (2, 7). A sulfur-containing intermediate produced by NFS1 is exported from the mitochondrion to the cytosol, before being combined with iron and assembled on a cytosolic scaffold protein complex, which contains NBP35 (7). This is followed by a number of extra carrier proteins that help insert the nascent cluster into Fe-S proteins in the cytosol and nucleus (Fig. 1A) called Nar1, CIA1, CIA2, and MMS19. Thus, the cytosolic CIA pathway requires mitochondrial export of sulfur-containing intermediate to function.

Mitochondrial export of this sulfur-containing intermediate is performed by an ATP-binding cassette (ABC) transporter called ABCB7 (ATM1 in yeast, ATM3 in plants), in a glutathione-dependent manner (7, 9). ABCB7 is a type IV ABC transporter (10) localized to the inner mitochondrial membrane, with a nucleotide-binding domain (NBD) facing the mitochondrial matrix, suggesting it functions as an exporter. ABCB7 transporters operate as half-transporters, with six transmembrane domains from each monomer contributing to forming the substrate binding cavity (11). Initial work in yeast identified ATM1 as the exporter required for cytosolic Fe-S cluster maturation (12). Subsequent studies in yeast, plants, and vertebrate models have shown a conserved function, where depletion of this ABC transporter leads to defects in cytosolic and nuclear Fe-S proteins, while the mitochondrial Fe-S proteins remain largely unaffected (13, 14). Recent structural studies in yeasts, plants, and mammals have helped elucidate the transport mechanism (15–19), although the exact physiological substrate of the transporter is still the subject of debate. Studies in plants suggest glutathione polysulfides as potential substrates (20), while another proposed glutathione-coordinated 2Fe-2S clusters (21). Multiple physiological substrates have also been proposed (22), with distinct Fe-and S-containing and S-only containing intermediates both being exported by the transporter and being used for distinct processes: the Fe- and S-containing intermediate for cytosolic Fe-S protein maturation and a distinct sulfur-containing intermediate being used for tRNA thiolation (23). While the exact nature of the physiological sulfur-containing substrate continues to be debated (24), the effect of ABCB7 depletion on the downstream cytosolic and nuclear Fe-S proteins is clear and conserved among commonly studied model organisms (11). However, despite its seeming universal importance, our understanding of how Fe-S cluster biosynthetic pathways work in the medically important apicomplexan parasites is incomplete.

Apicomplexa are obligate intracellular parasites that cause diseases of global importance, such as malaria and toxoplasmosis. *Toxoplasma gondii*, the causative agent of toxoplasmosis, can infect most warm-blooded animals and cause life-threatening diseases in the immunocompromised. As well as being an important pathogen, it is a versatile model organism for studying apicomplexan biology. Recent studies have begun to explore the understudied Fe-S biogenesis pathways in *Toxoplasma*. Studies looking at proteins in the CIA, ISC, and SUF pathways have shown their importance for parasite fitness (25–27). Interruption of the ISC pathway, by depleting the mitochondrial scaffold protein ISU1, was shown to have a severe, but reversible, impact on parasite fitness and to trigger partial stage conversion into bradyzoites, a cyst-enclosed persisting stage that is involved in the chronic phase of toxoplasmosis (25). The important relationship between the mitochondrion and cytosolic Fe-S biogenesis was further supported by a study that uncovered the unusual mitochondrial anchoring of the CIA scaffold protein NBP35, which was characterized as a cytosolic protein in other eukaryotes (27). This unusual finding highlights how little we understand about how Fe-S cluster biogenesis intersects with parasite metabolism despite its importance to their fitness and life cycle.

Here, we have sought to test whether cytosolic Fe-S protein biogenesis is dependent on the mitochondrial pathway in *Toxoplasma*, specifically if an ABCB7-type function exists, and determine the broader impact on parasite fitness of this connection between the mitochondrial and cytosolic pathways.

## RESULTS

### Does *Toxoplasma* encode an ABCB7-type transporter?

To identify potential functional equivalents of the mitochondrial transporter ABCB7 in *Toxoplasma*, we performed BLAST similarity searches on the ToxoDB.org database (28) using both yeast and human ABCB7 orthologs (ATM1, Uniprot: P40416; ABCB7, Uniprot: O75027). These searches identified *Toxoplasma* protein TGGT1_269000 as the top hit. Multiple sequence alignment showed conservation of key functional residues involved in glutathione and ATP binding (Fig. S1A) and structural predictions using Alphafold (29, 30) suggested overall structural conservation with an experimentally determined structure (Fig. S1B). While this gene had previously been annotated as an ABCB7 homolog (25), it had also been suggested to be a putative homolog of the closely related ABC transporter ABCB6 (29–31) . To test for resolvable relationships between TGGT1_269000 and characterized ABCB6 and ABCB7 proteins, we performed a phylogenetic analysis that included characterized ABCB6 and ABCB7 proteins and homologs sampled from major clades across the eukaryotic tree (Fig. 1B). This analysis recovered one strongly supported clade (bootstrap support = 97) containing characterized or well-studied ABCB6 homologs from model animals (human, mouse, rat, *Drosophila melanogaster,* and *Danio rerio*), along with proteins from diverse eukaryotic groups. Apicomplexans are represented in this ABCB6 clade including the *Toxoplasma* gene TGGT1_273360, providing strong evidence of a *Toxoplasma* ABCB6 ortholog other than TGGT1_269000 (Fig. 1B; Fig. S2). Conversely, while animal ABCB7 proteins formed a strongly supported clade, this was not within a larger supported clade with other eukaryotic groups, including the yeast putative ortholog ATM1. TGGT1_269000 belongs to a strongly supported clade with other myozozoans (apicomplexans, chrompodellids, squirmids, dinoflagellates, and perkinsids) (Fig. 1B; Fig. S2), but the position of this orthogroup is also not resolved other than being more closely related to ABCB6 and ABCB7 than any other ABCB paralogs that were included in the analysis as an outgroup (bootstrap support = 100).

Therefore, while TGGT1_269000 is revealed as the best candidate for the ABCB7-type function, experimental investigations were required to test this. We therefore named this protein ABCB7-like (ABCB7L) and proceeded to test its function.

### Biochemical characterization of *Tg*ABCB7L

To biochemically characterize *Tg*ABCB7L, we first created a C-terminal triple hemagglutinin (HA) epitope-tagged version of the endogenous protein using a CRISPR-mediated homologous recombination approach, outlined in Fig. 1C, in the TATiΔ*ku80* parental strain (32). A clonal line, named ABCB7L-HA, was isolated from a positive pool by serial dilution and confirmed by PCR (Fig. 1D). Immunoblot analysis of ABCB7L-HA showed a clear and specific signal, migrating above the 95 kDa molecular weight marker at approximately 105 kDa (Fig. 1E). This is slightly smaller than the predicted size of 125 kDa, likely due to cleavage of a mitochondrial targeting sequence, as seen in yeast and plant homologs (16, 18). ABCB7 forms homodimers to create functional transporters (11). We therefore performed native-PAGE and immunoblot analysis of proteins extracted from ABCB7L-HA parasites. Size estimation compared to membrane-bound bovine mitochondrial complexes suggested migration at 287 KDa, a size consistent with migration of the homodimer (Fig. 1F). Longer exposures show the presence of a second band migrating at 111 kDa, possibly showing migration of monomeric *Tg*ABCB7L that has not yet been incorporated in a homodimer (Fig. S3A). ABCB7L-HA parasites treated with SDS, a stronger detergent that breaks weaker protein-protein interactions, showed a single band migrating at the size of a monomer (Fig. S3B). To provide additional support for homodimer formation, we introduced a second, C-terminally Ty-epitope tagged, version of the protein and performed reciprocal co-immunoprecipitation (co-IP) experiments. Ty signal was detected in the bound fraction of the HA IP, and HA signal was detected in the bound fraction of the Ty IP, while no signal from the unrelated proteins TOM40 and CDPK1 was found in either bound fraction (Fig. S3C) indicating interaction between the two copies of the protein, and consistent with *Tg*ABCB7L homodimer formation.

Previous proteomic studies have suggested a mitochondrial localization for *Tg*ABCB7L. The protein was detected in a recent *Toxoplasma* organelle proteomic atlas using hyperLOPIT and was predicted to be in the mitochondrial membranes fraction (33). It was also detected in a proximity-labeling proteome of the mitochondrial matrix (34) and a complexome profiling analysis of *Toxoplasma* mitochondria (35). To confirm that *Tg*ABCB7L is mitochondrially localized, we performed an immunofluorescence assay (IFA). As predicted, the HA signal co-localized with the mitochondrial marker protein MYS (36), confirming mitochondrial localization (Fig. 1G).

The *Tg*ABCB7L sequence is predicted to have multiple transmembrane domains, using the prediction software TMHMM 2.0 (37), consistent with its function as a transporter. To provide support for *Tg*ABCB7L being a transmembrane protein, we performed sodium carbonate (Na_2_CO_3_) extractions. Resistance to Na_2_CO_3_ extraction can distinguish integral membrane proteins from soluble or periphery-associated membrane proteins (27, 38, 39). ABCB7L-HA was detected in the Na_2_CO_3_ extraction-resistant pellet fraction, along with the mitochondrial integral membrane protein TOM40 (Fig. 1H), while the cytosolic protein CDPK1 and the peripherally membrane-associated ATP synthase subunit, ATPβ, were soluble upon Na_2_CO_3_ treatment. To test that none of the proteins detected are inherently insoluble, samples were also subjected to extraction with the Triton X-100 (TX-100) detergent: as expected, in these conditions, all proteins were found in the soluble fraction, indicating membrane proteins were efficiently solubilized in protein-detergent mixed micelles (Fig. 1H). Taken together, these data are in support of *Tg*ABCB7L being an integral membrane protein, consistent with its role as a transporter.

### *Tg*ABCB7L is required for growth and its depletion leads to partial conversion into bradyzoites

**Fig 2:**
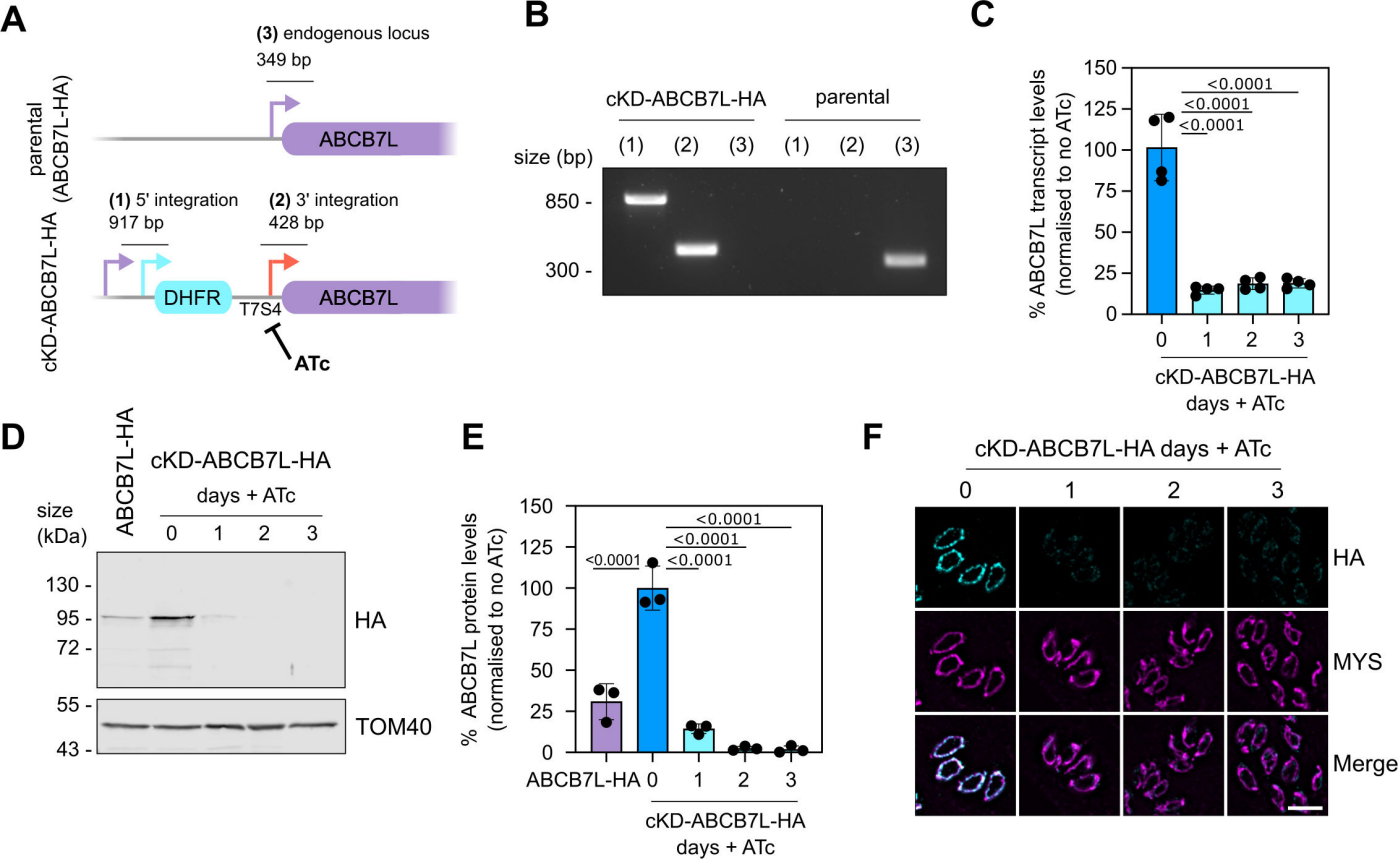
Replacement of *Tg*ABCB7L promoter with a regulatable promoter results in a tightly controlled conditional knockdown line. (**A**) A schematic of the promoter replacement strategy is used to create a conditional knockdown of *Tg*ABCB7L (cKD-ABCB7L-HA). The expected size of integration PCRs is shown. (**B**) PCR to test the integration of the DHFR selection cassette and the regulatable promoter into the endogenous locus, as outlined in panel **A**. (**C**) Relative transcript levels of *Tg*ABCB7L in cKD-ABCB7L-HA line after 1, 2, and 3 days Anhydrotetracycline (ATc) treatment, measured by qRT-PCR. Error bars are mean ± S.D., and a one-sample *t*-test is used to compare transcript levels in plus ATc to no ATc, which was set to one, *n* = 4. (**D**) Immunoblot analysis of whole-cell lysate extracted from cKD-ABCB7L-HA parasites treated with ATc for 0, 1, 2, and 3 days, and ABCB7L-HA parasites. Samples were separated by SDS-PAGE, blotted, and detected using anti-HA, to visualize ABCB7L-HA, and anti-TOM40 as a loading control. (**E**) Quantification of immunoblots in panel **D**. Each point represents a replicate, normalized to TOM40, and the mean of zero-day ATc is set at 100%. Error bars are mean ± SD, protein levels were compared to cKD-ABCB7L-HA without ATc using one-way ANOVA, with a Dunnett correction for multiple comparisons, *n* = 3. (**F**) Immunofluorescence assay analysis of cKD-ABCB7L-HA parasites treated with ATc for 0, 1, 2, and 3 days, labeled with anti-HA to detect ABCB7L-HA protein (cyan), and MYS as a mitochondrial marker (magenta). The scale bar is 5 µm.

We next wanted to study the role of *Tg*ABCB7L in *Toxoplasma* growth and Fe-S cluster metabolism. Data from a previous *Toxoplasma* genome-wide CRISPR screen (40), assigned *Tg*ABCB7L a phenotype score of –4.96, indicating that it is likely essential for the fitness of tachyzoites (the fast-replicating stage of the parasite responsible for the acute phase of toxoplasmosis) under standard culture conditions. Therefore, to test the role of this protein, we generated a conditional knockdown line by replacing the native promoter with an anhydrotetracycline (ATc) regulated promoter (32, 41) using a CRISPR-mediated homologous recombination approach, in the ABCB7L-HA background, outlined in Fig. 2A. A clonal line, named cKD-ABCB7L-HA, was isolated from a positive pool by serial dilution and confirmed by PCR (Fig. 2B). To assess the extent of downregulation of *Tg*ABCB7L transcript upon the addition of ATc, we performed qRT-PCR on parasites grown in the absence or presence of ATc for one, two, and three days. Parasites grown in ATc showed significantly lower levels of the transcript, confirming controlled depletion (Fig. 2C). To assess the effect of depletion of the transcript on *Tg*ABCB7L protein levels, we performed immunoblot analysis on parasites grown in the absence or presence of ATc for one, two, and three days. The ABCB7L-HA protein was decreased after one day in ATc, and virtually undetectable after two and trhee days (Fig. 2D and E). Similar results were seen with IFA analysis (Fig. 2F). Notably, the levels of ABCB7L-HA protein were ~four times increased in the conditional knockdown line compared to the parental ABCB7L-HA line, likely due to mis-regulation by inserting a non-native promoter (Fig. 2D and E). However, quantification of immuno-signal shows that after the addition of ATc, protein levels are decreased compared to the parental cell line (Fig. 2E).

**Fig 3:**
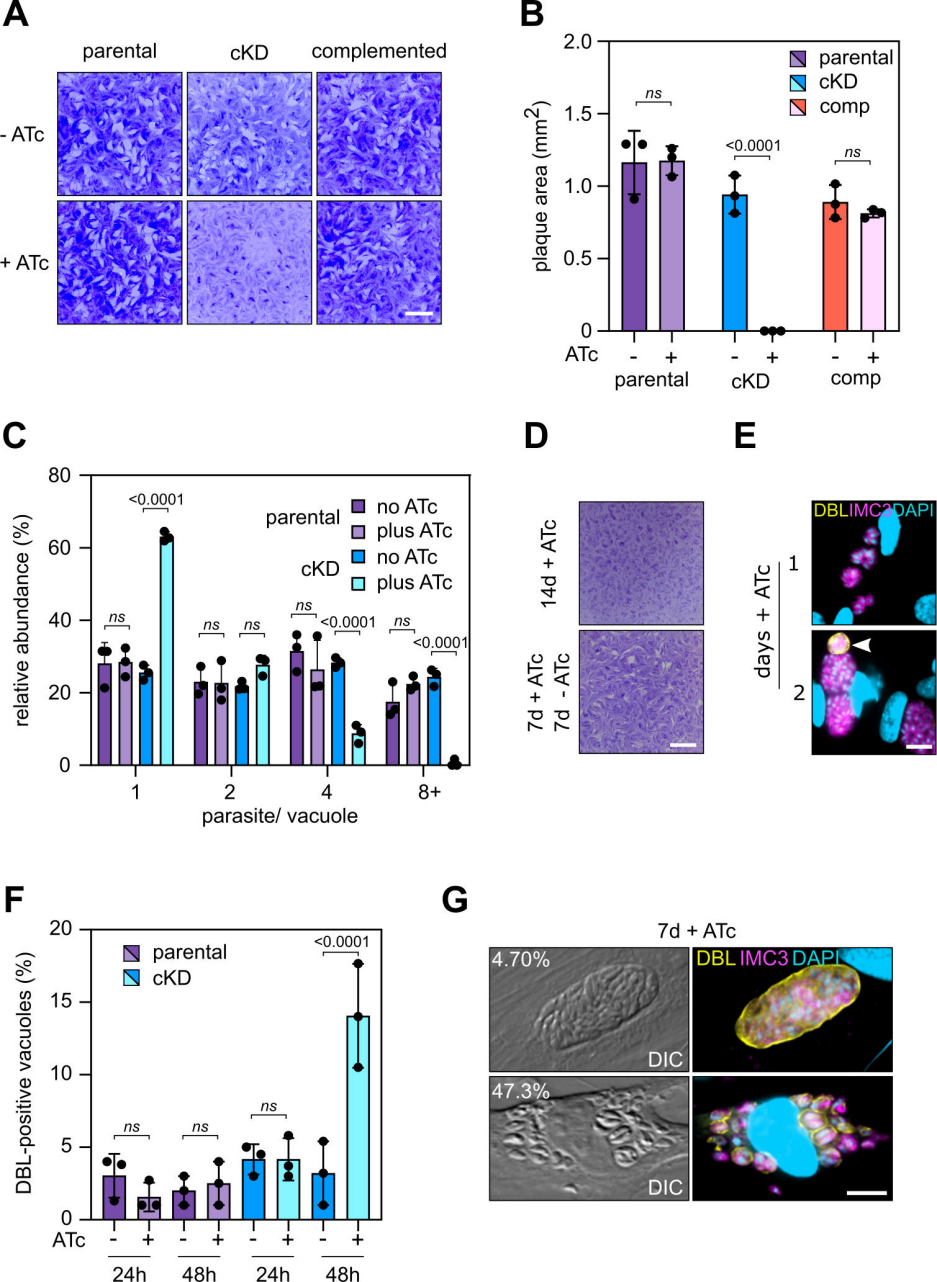
Downregulation of *Tg*ABCB7L leads to a decrease in parasite replication and triggers partial parasite differentiation. (**A**) Plaque assays of parental, cKD-ABCB7L-HA, and complemented parasites, grown in the presence or absence of ATc for 8 days. Scale bar is 5 mm (**B**) Quantification of plaque assays from panel **A**. Three independent experiments were performed, and 50 plaques were measured per replicate. The mean of each replicate was displayed, ±SD. One-way ANOVA followed by Tukey’s multiple pairwise comparisons was performed, and the *P*-value from relevant pairs is displayed. (**C**) Quantification of a number of parasites per vacuole for parental and cKD-ABCB7L-HA parasites. Parasites were grown in the presence or absence of ATc for 2 days before inoculation into fresh HFFs cells and grown for a further day in the presence or absence of ATc. Error bars are mean ± SD from three independent experiments, for which over 100 vacuoles were counted for each replicate. One-way ANOVA followed by Tukey’s multiple pairwise comparisons was performed, and the *P*-value from relevant pairs was displayed. (**D**) Plaque assays of cKD-ABCB7L-HA parasites, grown for either 14 days in the presence of ATc or 7 days in ATc followed by an ATc washout, followed by growth for 7 days in the absence of ATc. The scale bar is 5 mm. (**E**) Immunofluorescence assay of cKD-ABCB7L-HA parasites treated with ATc for 1 or 2 days, labeled with IMC3 (magenta) to detect parasites and a lectin of *Dolicos biflorus* (DBL) to outline nascent cyst walls. 4′,6-diamidino-2-phenylindole (DAPI) was used to stain DNA. The scale bar is 10 µm. (**F**) Quantification of DBL-positive vacuoles from parental and cKD-ABCB7L-HA parasites treated with ATc for 1 or 2 days. Data are from *n* = 3 independent experiments. One hundred vacuoles were counted per experiment. Values are mean ± SD. One-way ANOVA followed by Tukey’s multiple pairwise comparisons was performed, and the *P*-value from relevant pairs was displayed. (**G**) Immunofluorescence assay of cKD-ABCB7L-HA parasites treated with ATc for 7 days, labeled with anti-IMC3 (magenta) to detect parasites and DBL to outline nascent cyst walls (yellow). The percentage at the top of the differential interference contrast (DIC) image represents the proportion of vacuoles that are DBL-stained and resemble mature cysts (top) or are DBL-stained but with less than four parasites (bottom), data are from *n* = 3 independent experiments in which at least 100 vacuoles were counted. DAPI was used to stain DNA. The scale bar is 10 µm.

The ABCB7 transporter is functionally required for the Fe-S cluster biosynthesis pathways in other species (7) and, combined with the low phenotype score (40), *Tg*ABCB7L is predicted to be important for *Toxoplasma* fitness. To test this, we performed plaque assays. The cKD-ABCB7L-HA line was grown on an HFF monolayer for eight days and was unable to form plaques in the presence of ATc (Fig. 3A), confirming the importance of *Tg*ABCB7L for parasite growth *in vitro*. The cKD-ABCB7L-HA parasites formed slightly smaller plaques than the parental control (Fig. 3A and B), suggesting a small fitness consequence of the insertion of the regulatable promoter, possibly due to increased *Tg*ABCB7L protein levels (Fig. 2D and E). To show that the observed phenotype is due to the depletion of *Tg*ABCB7L protein, we engineered a line containing a second Ty epitope-tagged copy of *Tg*ABCB7L under the TUB8 promoter (42), in the conditional knockdown line, which we named cKD-ABCB7L-HA/ABCB7L-Ty. IFA analysis showed this second copy is mitochondrially localized (Fig. S4A). Immunoblot analysis shows that after growing cKD-ABCB7L-HA/ABCB7L-Ty in the presence of ATc for three days, the ATc regulatable HA-tagged version of the protein is depleted, but the Ty-epitope tagged version is unaffected (Fig. S4B). We performed plaque assays with the cKD-ABCB7L-HA/ABCB7L-Ty line that showed no growth defect after eight days in ATc, demonstrating phenotypic rescue (Fig. 3A and B).

To further characterize the effect of *Tg*ABCB7L depletion on parasite growth, we grew parental and cKD-ABCB7L-HA parasites in the presence or absence of ATc for two days, then allowed parasites to invade an HFF monolayer, grow for a further 24 hours, and then performed an IFA to assess parasite replication. We quantified the number of parasites per vacuole and found a significant decrease in the number of vacuoles containing four and eight (or more) parasites, and an increase in one parasite vacuoles, in the cKD-ABCB7L-HA cell line grown in ATc (Fig. 3C). All other conditions resulted in similar numbers of parasites per vacuole. These data further support a marked impact on parasite replication when *Tg*ABCB7L is depleted.

Our previous study looking at the mitochondrial Fe-S scaffold ISU1 (25) discovered a reversible growth defect when ISU1 is depleted. To assess whether this was also the case in our *Tg*ABCB7L depletion mutant, we grew cKD-ABCB7L-HA parasites in ATc for 14 days or ATc for seven days, followed by ATc wash-out and a further seven days in normal media. The parasites grown in ATc for 14 days formed no plaques, whereas the parasites where the ATc was washed out formed small plaques (Fig. 3D). This suggests that, similar to what was observed for the ISU1 mutant, cKD-ABCB7L-HA parasites are still viable after seven days of protein depletion and the growth defect is partially reversible, consistent with a functional connection between these two pathways.

We have previously shown that disruption of mitochondrial functions, including mitochondrial Fe-S biosynthesis, led to partial differentiation into bradyzoites and started forming cyst-like structures (25, 39). To see whether this was also the case for the *Tg*ABCB7L conditional knockdown line, we performed an IFA, using a lectin from *Dolichos biflorus* (DBL), which labels the cyst-wall glycoprotein CST1 that is built up during tachyzoite to bradyzoite conversion (43). We observed numerous DBL-positive structures (Fig. 3E), and quantification showed they were significantly increased in cKD-ABCB7L-HA parasites grown in ATc for two days, with ~15% of DBL positive vacuoles (Fig. 3F). Long-term *in vitro* cultured cysts are usually large structures containing dozens of bradyzoites. To see whether *Tg*ABCB7L depletion led to mature cysts, we performed DBL imaging after seven days of incubation with ATc. More than half (52%) of the vacuoles showed DBL staining, yet the vast majority of these (~47%) contained four or fewer parasites, whilst only ~5% formed large, disorganized, cyst-like structures (Fig. 3G). These data suggest that, similar to what is seen in mitochondrial mutants, depletion of *Tg*ABCB7L leads to the initiation of stage conversion, but this process is likely not completed.

Overall, our data show that *Tg*ABCB7L is essential for parasite growth *in vitro* and that its depletion leads to the initiation of partial-stage conversion.

### Mitochondrial functions are unaffected after *Tg*ABCB7L downregulation

**Fig 4:**
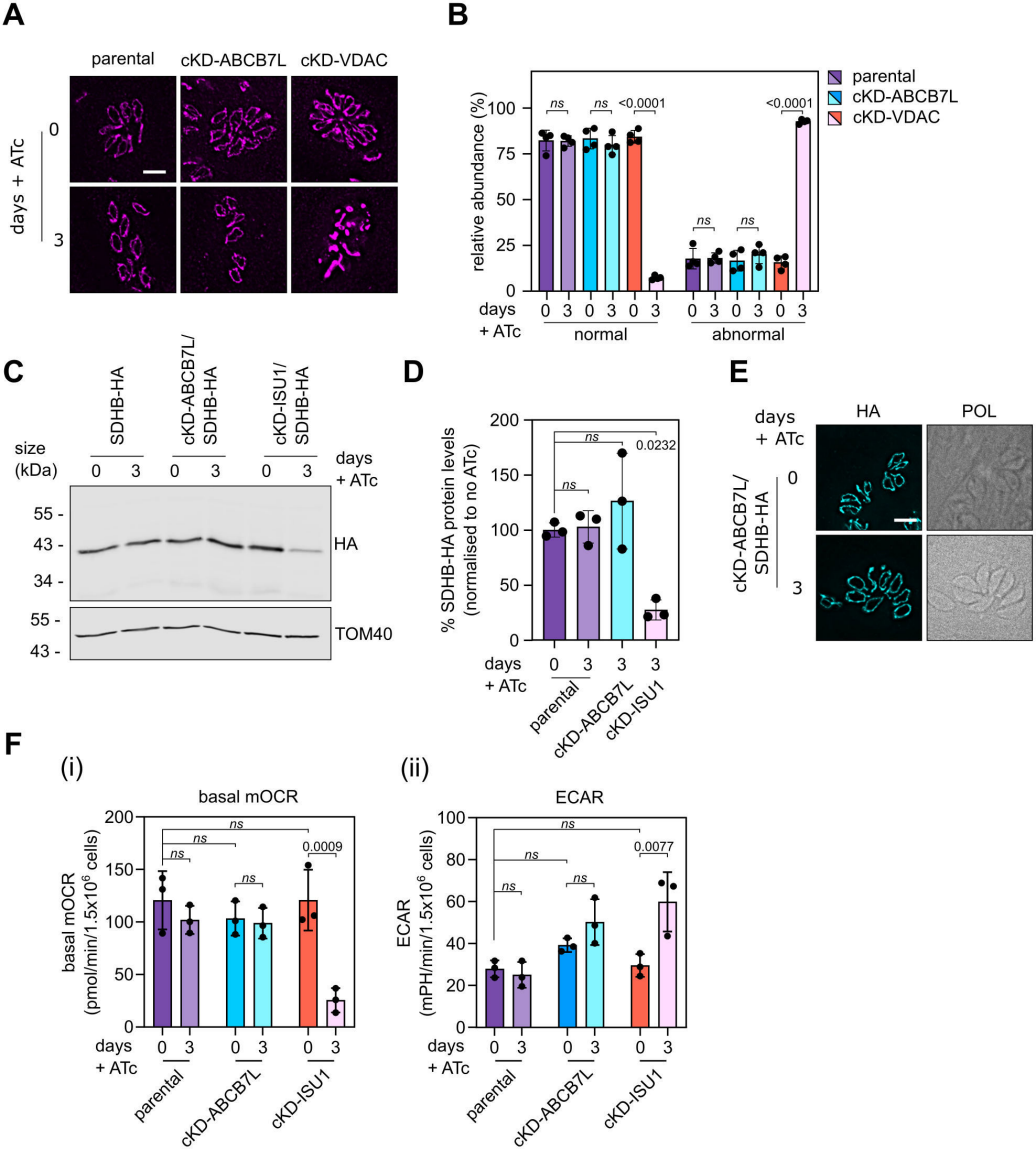
Mitochondrial functions are unaffected after downregulation of *Tg*ABCB7L. (**A**) Immunofluorescence assay analysis of parental, cKD-ABCB7L-HA, and cKD-VDAC-HA parasites grown in the presence or absence of ATc for 3 days, labeled with TOM40 to visualize mitochondrial morphology. (**B**) Quantification of mitochondrial morphology from parental, cKD-ABCB7L-HA, and cKD-VDAC lines at 3 days in the presence or absence of ATc. Morphologies were scored as normal or abnormal. Error bars are mean ± SD from four independent experiments, for which over 150 vacuoles were counted for each replicate. One-way ANOVA followed by Tukey’s multiple pairwise comparisons was performed, and the *P*-value from relevant pairs was displayed. (**C**) Immunoblot analysis of whole-cell lysate extracted from SDHB-HA, cKD-ABCB7L/SDHB-HA, and cKD-ISU1/SDHB-HA parasites grown in the presence or absence of ATc for 3 days. Samples were separated by SDS-PAGE, blotted, and detected using anti-HA, to visualize SDHB-HA, and anti-TOM40 as a loading control. (**D**) Quantification of immunoblots in panel **C**. Each point represents a replicate, normalized to TOM40, and the mean of parental zero-day ATc was set at 100%. Error bars are mean ± SD from three independent experiments. One-way ANOVA followed by Tukey’s multiple pairwise comparisons was performed, and the *P*-value from relevant pairs was displayed, *n* = 3. (**E**) Immunofluorescence assay analysis of SDHB-HA and cKD-ABCB7L/ SDHB-HA parasites grown in the presence or absence of ATc for 3 days, labeled with anti-HA to detect SDHB-HA protein. The scale bar is 5 µm. (**F**) Extracellular flux analysis, using a seahorse analyzer, basal mitochondrial oxygen consumption rate (OCR), and extracellular acidification rate (ECAR) of parental, cKD-ABCB7L-HA, and cKD-ISU1-HA parasites grown in the presence or absence of ATc for 3 days. Graphs show mean ± SD from three independent experiments. One-way ANOVA followed by Tukey’s multiple pairwise comparisons was performed, and the *P*-value from relevant pairs was displayed.

Numerous studies in other model systems show that mutants in ABCB7 function lead to defects in cytosolic and nuclear Fe-S proteins, while mitochondrial Fe-S proteins remain unaffected. If this is the case in *Toxoplasma* too, we would expect mitochondrial biology to remain unaffected. To address this hypothesis, we first analyzed general mitochondrial morphology upon depletion of *Tg*ABCB7L. Typically, intracellular parasites have a single mitochondrion which is predominantly found in a “lasso” shape that spans the parasite periphery. Our previous studies have shown that some defects in mitochondrial biology can result in alteration of this morphology (41, 44). We performed IFAs on parasites grown in the absence or presence of ATc for three days from parental and cKD-ABCB7L-HA lines, as well as cKD-VDAC-HA, a conditional knockdown line of the mitochondrial transporter VDAC, which has a severe defect in mitochondrial morphology (44) and thus used as a positive control. This analysis showed in cKD-ABCB7L-HA parasites, normal “lasso”-shaped mitochondria can still be formed after three days in ATc, in contrast to cKD-VDAC-HA which has almost exclusively abnormal (i.e., shortened or collapsed) mitochondria (Fig. 4A and B).

Previous studies in *Toxoplasma* have shown that defects in the mitochondrial Fe-S biosynthetic pathway (the ISC pathway), either through depletion of the cysteine desulfurase NFS1 (27) or the scaffold protein ISU1 (25), lead to a decrease in the abundance of the Fe-S cluster containing succinate dehydrogenase (mETC complex II) subunit SDHB. Given that in other systems, depletion of ABCB7 does not affect the mitochondrial ISC pathway, we expect a similar result here and for SDHB to be unaffected. To test this hypothesis, we generated a conditional knockdown line of *Tg*ABCB7L, as before, but now in a background where SDHB is C-terminally HA epitope tagged (35) (Fig. S5A) and confirmed *Tg*ABCB7L transcript depletion and growth arrest upon the addition of ATc (Fig. S5B and C). Immunoblot analysis shows that depletion of ABCB7 does not result in a decrease in SDHB protein while depleting ISU1 results in a ~70% decrease in SDHB protein (Fig. 4C and D), as seen previously (25). IFA analysis also shows no decrease or change in localization of SDHB protein levels (Fig. 4E). Together, these data show that depletion of *Tg*ABCB7L does not decrease SDHB protein abundance, in sharp contrast to the decrease observed upon depletion of ISC pathway members ISU1 or NFS1.

One of the main clients of mitochondrially derived Fe-S clusters is the mETC, where complexes II, III, and IV in *Toxoplasma* all contain subunits predicted to contain clusters (25, 34, 35, 45, 46). Depletion of NFS1 or ISU1 leads to a decrease in mETC capacity (25, 27). To test whether depletion of *Tg*ABCB7L affects the mETC, we measured the basal mitochondrial oxygen consumption rate (basal mOCR), using a seahorse extracellular flux analyzer. The basal mOCR has previously been used in *Toxoplasma* as a proxy measurement for mETC activity (34, 39, 45, 47), and mutants in the mitochondrial Fe-S pathway have been shown to have a lower mOCR (27). We observed no decrease in basal mOCR in the cKD-ABCB7L-HA line grown in ATc for three days (Fig. 4Fi), again suggesting no defect in the mETC. The parental line was also unaffected upon ATc treatment, but growth in ATc resulted in a significant decrease in basal mOCR in the cKD-ISU1-HA line as expected. We also measured the extracellular acidification rate (ECAR), as a proxy for general parasite metabolism. We observed no defect in ECAR, showing that the parasites are still viable (Fig. 4Fii). ECAR in cKD-ISU1-HA was increased, perhaps due to a compensatory increase in glycolysis.

These data suggest that depletion of *Tg*ABCB7L does not affect the important mitochondrial functions that Fe-S proteins are involved in, and thus likely the biosynthesis of mitochondrial Fe-S proteins is unaffected. This suggests that the observed growth and differentiation defects of the cKD-ABCB7L-HA mutant likely involve proteins or pathways that act downstream of the mitochondrion.

### Label-free quantitative proteomics identifies pathways affected by *Tg*ABCB7L depletion

**Fig 5:**
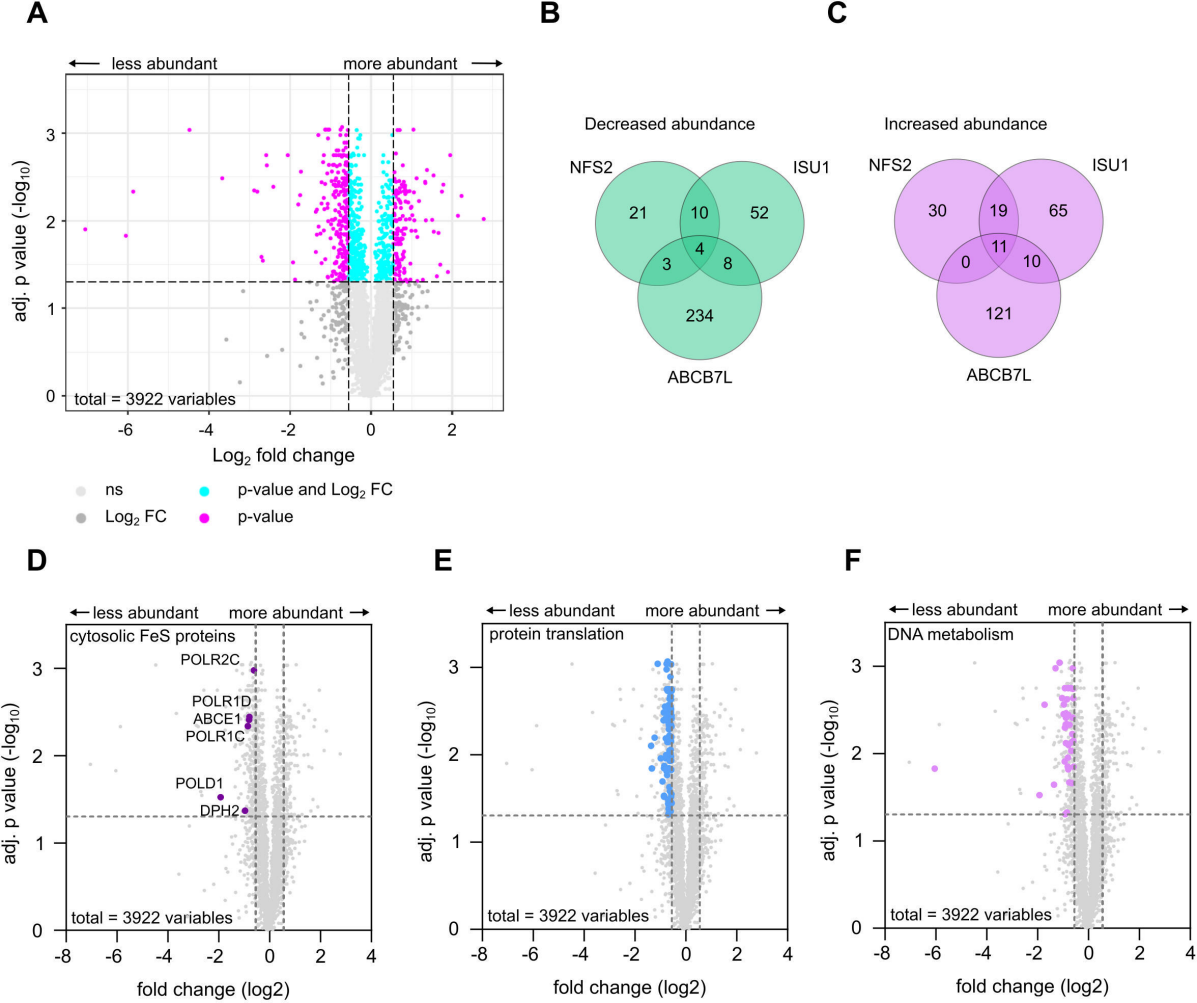
*Tg*ABCB7L depletion impacts cytosolic Fe-S proteins and proteins involved in DNA metabolism and translation. (**A**) Volcano plot showing the difference in protein abundance, determined by label-free quantitative proteomic data, in cKD-ABCB7L-HA parasites grown in ATc for 3 days. The *y*-axis shows the Log_2_ fold change compared to the parental line grown in the same conditions, and the *x*-axis shows the −log10(*P* value) after a Student’s *t*-test with a Benjamini correction for multiple comparisons applied, for four independent biological replicates. (**B** and **C**) Venn diagrams depicting shared and unique proteins whose abundance is affected by the depletion of *Tg*ABCB7L, and NFS2 and ISU1 from reference (25). (**D**) Volcano plot of LFQ data with cytosolic Fe-S proteins showing decreased abundance labeled. (**E, F**) Volcano plot of LFQ data with proteins involved in protein translation (**E**) and DNA metabolism (**F**) showing decreased abundance labeled.

Having shown the importance of *Tg*ABCB7L for *Toxoplasma* fitness, we asked what global protein changes occurred when *Tg*ABCB7L was depleted, to see whether these could identify how the transporter is integrated into the biosynthesis of FeS clusters and the effects on wider parasite metabolism. Our previous study on the importance of the ISC and SUF pathway on parasite fitness used label-free quantitative (LFQ) proteomics to assess the effect of disrupting specific proteins in the pathways on the global parasite proteome (25). We performed a similar analysis on parental and cKD-ABCB7L-HA parasites grown in ATc for three days. Total protein samples from four independent experiments were subjected to mass spectrometry analysis, and LFQ values were calculated to compare protein levels between parental and knockdown parasites. In total, 3,922 proteins were detected at high enough quality to calculate LFQ intensity values. The *Tg*ABCB7L protein was detected in all parental replicates, but was undetected in all mutant replicates, confirming it is efficiently downregulated below the detection threshold. In all, 140 proteins were significantly increased in abundance (log2[fold change ]≥0.55) in the ckD-ABCB7-HA line compared to parental, while 249 were of lower abundance (log2[fold change] ≤ −0.55) (Tables S1 and S2). There was little overlap in the identity of the proteins increased or decreased in abundance compared to similar analysis on the mitochondrial Fe-S scaffold ISU1 (Fig. 5B) or the apicoplast NFS2 (Fig. 5C) (25), suggesting the pattern of protein abundance change is specific to the depletion of *Tg*ABCB7L. Analysis of the predicted localization of decreased proteins through the *Toxoplasma* hyperLOPIT-based organelle proteomic data set (33) shows a significant increase in the proportion of proteins resolving to the 40S and 60S ribosome clusters (Table S3).

To investigate in more detail the effect of the depletion of *Tg*ABCB7L, we looked in our proteomics data set for components of the ISC and CIA pathways. We did not observe a strong effect on the proteins of the CIA pathways, with none of its members increased or decreased in abundance above the ≥0.55 threshold (Fig. S5A). This suggests that *Tg*ABCB7L depletion has little impact on the CIA pathway abundance. Interestingly, in the mitochondrial ISC pathway, two proteins involved in the early steps of sulfur generation, the cysteine desulfurase NFS1 and ISD11, were increased in abundance, as well as a glutaredoxin (Fig. S6A), perhaps upregulated as part of a feedback loop to compensate for the lack of sulfur-containing intermediate in the cytosol.

We previously predicted non-organellar Fe-S proteins using the Fe-S protein prediction software MetalPredator (25, 48). We manually updated this to include proteins predicted to be organellar in the *Toxoplasma* hyperLOPIT data set (33), but which have experimental evidence to be cytosolic facing proteins, for example, NBP35 (27), RlmN (49), and ELP3 (50). In the absence of a functional cluster, the Fe-S cluster containing proteins may be degraded (51). 18 of the 28 proteins in this list were detected at high enough quality to calculate LFQ intensity values. Six of these were of decreased abundance, below the fold change cut-off (Table 1; Fig. 5D; Fig. S6B). These include Fe-S cluster containing subunits of DNA and RNA polymerases, as well as factors important for translation. In addition, the predicted Fe-S cluster containing DNA primase large subunit, TGGT1_297840 (PRIM2), and the DNA polymerase family B protein, TGGT1_319860 (POLE), were detected in all four parental replicates, but not in all replicates of the *Tg*ABCB7L knockdown sample (Table 1; Tables S1 and S2). By contrast, none of the predicted mitochondrial or apicoplast Fe-S proteins were found to be decreased upon *Tg*ABCB7L depletion (Fig. S6C). These data suggest that a depletion of ABCB7 leads to a preferential decrease in the abundance of non-organellar Fe-S proteins, as expected when the cytosolic biosynthetic pathway is disrupted.

**TABLE 1:** Cytosolic and nuclear Fe-S proteins decreased in abundance upon ABCB7L depletion

Gene ID	Name	Phenotype score^*a*^	Localization (hyperLOPIT)^*b*^	Function
TGGT1_258030	POLD1	−4.45	Nucleolus	Subunit of DNA polymerase delta complex with role in DNA replication
TGGT1_238190	POLR1C	−4.42	Nucleus—non-chromatin	Subunit of DNA-directed RNA polymerases I and III
TGGT1_261540	POLR1D	−3.54	Nucleus—non-chromatin	Subunit of DNA-directed RNA polymerases I and III
TGGT1_267390	POLR1C	−5.2	Nucleus—non-chromatin	Subunit of DNA-directed RNA polymerases I and III
TGGT1_216790	ABCE1	−4.11	Nucleus—non-chromatin	Ribosome recycling factor
TGGT1_261060	DPH2	−1.94	No data	Enzyme required for diphthamide biosynthesis; a post-translational modification important for translational fidelity
TGGT1_297840	PRIM2	−4.86	No data	Subunit of DNA primase and DNA polymerase alpha complex with role in DNA synthesis
TGGT1_319860	POLE1	−3.85	No data	Subunit of DNA polymerase epsilon complex with role in DNA replication

^*a*^ From genome-wide CRISPR screen (40).

^*b*^ From proteomic atlas using hyperLOPIT (33).

*Toxoplasma* is predicted to possess 14 proteins that use heme as a cofactor (31, 52), as well as a dedicated heme biosynthesis pathway composed of eight proteins (53). A previous study demonstrated two heme-containing proteins (cytochrome *c*-A and B) were decreased in abundance in a mutant of the biosynthetic protein UroD (54), suggesting that failure to assemble the heme cofactor can lead to decreases in protein abundance. No heme-containing or heme biosynthetic proteins were of decreased abundance in our mutant, and a single heme-containing protein, cytochrome *b*5-7 was of increased abundance (Fig. S6D). These data suggest that disruption of *Tg*ABCB7L has no effect on heme-protein abundance.

To look at the effect of *Tg*ABCB7L depletion on parasite biology, we further inspected the LFQ proteomic data, looking for pathways that were decreased in abundance. A gene ontology enrichment analysis (ToxoDB) of the 249 proteins decreased in abundance indicated that proteins involved in translation (59 proteins, 6.31-fold enrichment, *P*-value 2.5 × 10^−33^), ribosome biogenesis (10 proteins, 3.93-fold enrichment, *P*-value 1.78 × 10^−4^), DNA replication (nine proteins, 4.39-fold enrichment, *P*-value 1.59 × 10^−4^), DNA replication initiation (six proteins, 16.26-fold enrichment, *P*-value 3.34 × 10^−7^), and DNA-dependent DNA replication (six proteins, 8.61-fold enrichment, *P*-value 3.73 × 10^−5^) were significantly enriched in our data set. Further investigation of poorly annotated proteins in our data set using HHpred and domain searches found more factors involved in protein translation, ribosome and tRNA biogenesis, DNA and RNA replication, and metabolism. In total, we found 133 proteins that were significantly decreased below the fold change cut-off with homology to known components of these processes (Fig. 5G and H; Tables S1 and S2) consistent with experimental observations from other organisms (55).

### *Tg*ABCB7L depletion leads to a decrease in the DNA polymerase subunit POLD1 and the ribosome biogenesis factor ABCE1

**Fig 6:**
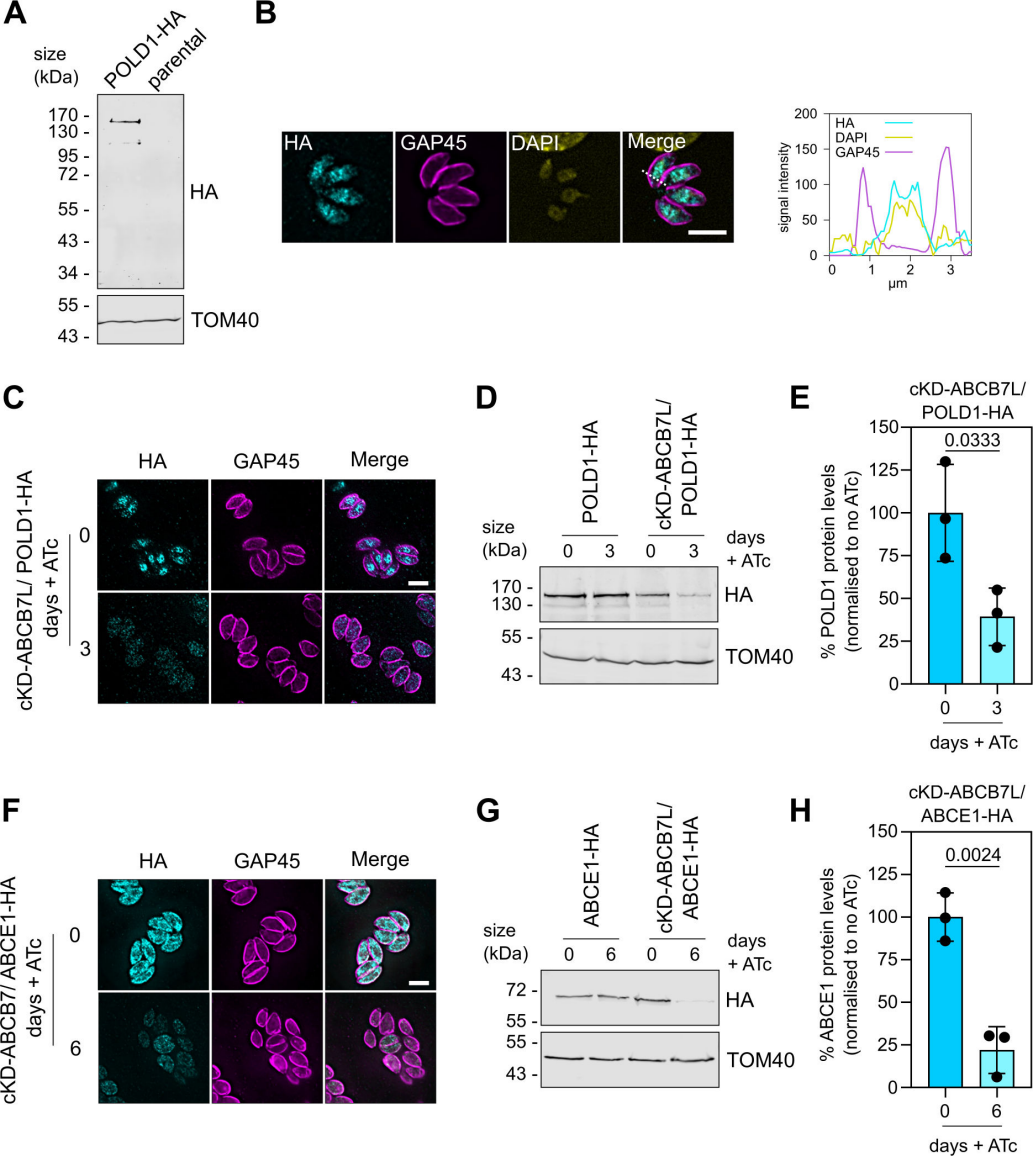
*Tg*ABCB7L depletion impacts POLD1 and ABCE1 protein levels. (**A**) Immunoblot analysis of whole-cell lysate extracted from POLD1-HA and parental parasites. Samples were separated by SDS-PAGE, blotted, and detected using anti-HA, to visualize POLD1-HA, and anti-TOM40 as a loading control. (**B**) Immunofluorescence assay analysis of POLD1-HA parasites, labeled with anti-HA to detect POLD1-HA (cyan), anti-GAP45 (magenta) to outline the parasites, and DAPI stain to label the nucleus. The scale bar is 5 µm. The graph shows the signal intensity for all three channels along the dotted line depicted in the merged image. (**C**) Immunofluorescence assay analysis of cKD-ABCB7L/ POLD1-HA parasites, grown in the presence or absence of ATc for 3 days, labeled with anti-HA to detect POLD1-HA (cyan), anti-GAP45 to outline the parasites (magenta). The scale bar is 5 µm. (**D**) Immunoblot analysis of whole-cell lysate extracted from POLD1-HA and cKD-ABCB7L/POLD1-HA parasites grown in the presence or absence of ATc for 3 days. Samples were separated by SDS-PAGE, blotted, and detected using anti-HA, to visualize POLD1-HA, and anti-TOM40 as a loading control. (**E**) Quantification of cKD-ABCB7L/ POLD1-HA immunoblots in panel **D**. Each point represents a replicate, normalized to TOM40, and cKD-ABCB7L/POLD1-HA the mean of zero-day ATc set at 100%. Error bars are mean ± SD, and an unpaired *t*-test was used to compare protein levels in plus and no ATc , *n* = 3. (**F**) Immunofluorescence assay analysis of cKD-ABCB7L/ ABCE1-HA parasites, grown in the presence or absence of ATc for 6 days, labeled with anti-HA to detect ABCE1-HA (cyan), anti-GAP45 to outline the parasites (magenta). The scale bar is 5 µm. (**G**) Immunoblot analysis of whole-cell lysate extracted from ABCE1-HA and cKD-ABCB7L/ ABCE1-HA parasites grown in the presence or absence of ATc for 6 days. Samples were separated by SDS-PAGE, blotted, and detected using anti-HA, to visualize ABCE1-HA, and anti-TOM40 as a loading control. (**H**) Quantification of cKD-ABCB7L/ABCE1-HA immunoblots in panel **G**. Each point represents a replicate, normalized to TOM40, and cKD-ABCB7L/ABCE1-HA zero-day ATc set at 100%. Error bars are mean ± SD, and an unpaired *t*-test was used to compare protein levels in plus ATc to no ATc, *n* = 3.

To validate our proteomic data, we performed focused experiments on two Fe-S cluster containing proteins that were significantly decreased. One of the most decreased proteins was the Fe-S cluster containing DNA polymerase delta catalytic subunit POLD1 (TGGT1_258030). POLD1 in humans has previously been shown to interact with the CIA-targeting complex and depend on MMS19 for cluster insertion (55). To study the effect of *Tg*ABCB7L depletion on POLD1, we created a C-terminal triple HA epitope-tagged version of the endogenous protein, using the same strategy as previously described and as outlined in Fig. S5D and E and named the line POLD1-HA. Immunoblot analysis of POLD1-HA showed a clear and specific band (Fig. 6A), migrating between the 130 and 170 kDa molecular weight marker, consistent with the predicted size of 143 kDa. IFA showed a diffuse signal across the cell, with an area of high signal overlapping with DAPI (Fig. 6B). Next, we created a conditional knockdown line of *Tg*ABCB7L in the POLD1-HA background (Fig. S5F), named cKD-ABCB7L/POLD1-HA and confirmed *Tg*ABCB7L transcript depletion and growth arrest upon the addition of ATc (Fig. S5G and H). To test whether *Tg*ABCB7L depletion affects POLD1-HA protein levels, we performed an IFA on cKD-ABCB7L/POLD1-HA, grown in the presence or absence of ATc for three days. HA signal was severely decreased, and largely absent from the nucleus, in the parasites grown in ATc (Fig. 6C). Immunoblot analysis also showed a decrease in POLD1 protein level in cKD-ABCB7L/POLD1-HA parasites grown in ATc (Fig. 6D and E). These data suggest that depletion of *Tg*ABCB7L leads to a decrease in POLD1 protein levels, consistent with the requirement of *Tg*ABCB7L for cytosolic Fe-S cluster maturation.

We next performed similar experiments on the Fe-S cluster containing ribosome recycling factor ABCE1, which has previously been shown to decrease in abundance upon disruption of the CIA pathway (27) and was found to be decreased in our proteomic data. We created a ABCE1-HA line (Fig. S5I and J) and showed similar protein size by immunoblot and localization by IFA as previously published (Fig. S5K and L) (27). We engineered a conditional knockdown line of ABCB7 in the ABCE1-HA background (Fig. S5M) and confirmed *Tg*ABCB7L transcript depletion and growth arrest upon the addition of ATc (Fig. S5N and O). To test whether *Tg*ABCB7L depletion affects ABCE1-HA protein levels, we performed IFA and immunoblot analysis on cKD-ABCB7L/ABCE1-HA, grown in the presence or absence of ATc. After three days in ATc, a slight decrease in ABCE1-HA levels in some cells could be detected by IFA (Fig. S5P). No detectable difference could be observed by immunoblot (Fig. S5Q). This may be due to the increased sensitivity of our proteomics approach compared to immunoblot analysis. To investigate further, we performed the same experiments at a later (six-day) time point of incubation with ATc, where we saw that the ABCE1-HA signal was severely decreased (Fig. 6F, G, and H). As a control, we performed the same experiment on cKD-ABCB7L/SDHB-HA parasites at six-day ATc growth and saw no decrease in SDHB-HA signal (S5R Fig), indicating the decrease is specific for ABCE1 and does not reflect general proteolysis due to a loss of viability, and is consistent with the parasites remaining viable for an extended period upon *Tg*ABCB7L depletion (Fig. 3C and H). Taken together, these experiments confirm POLD1 and ABCE1 decrease in abundance after *Tg*ABCB7L depletion, providing validation of our LFQ proteomic data set and providing support for *Tg*ABCB7L’s role in cytosolic Fe-S maturation.

### *Tg*ABCB7L depletion results in a global decrease in cytosolic translation

**Fig 7:**
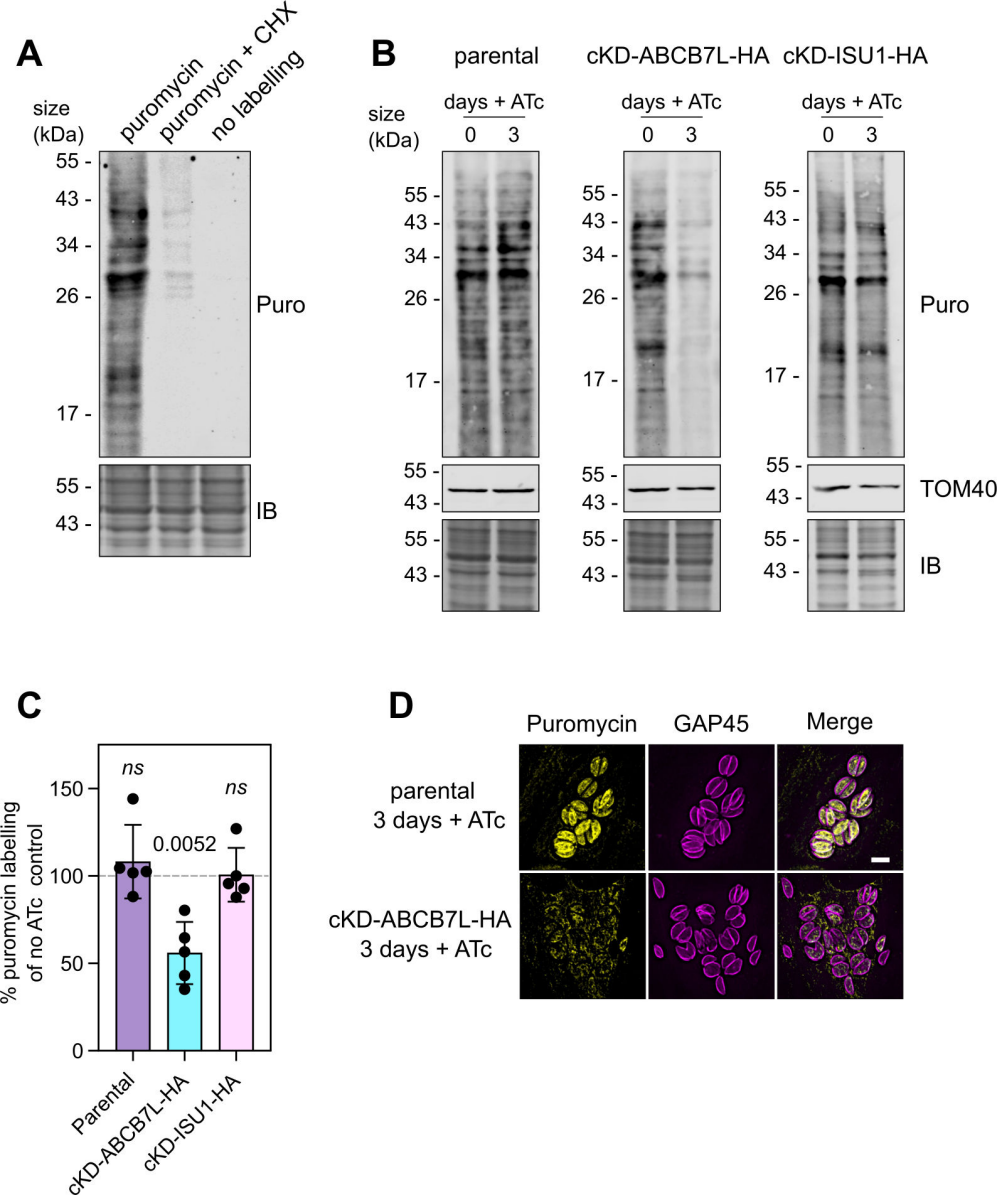
Cytosolic translation is decreased after depletion of *Tg*ABCB7L. (**A**) Immunoblot analysis of whole-cell lysate incubated with (i) puromycin for 15 minutes, (ii) cycloheximide for 10 minutes, followed by puromycin for 15 minutes, and (iii) no treatment. Samples were separated by SDS-PAGE, blotted, and detected using anti-puromycin to detect puromycin incorporation (top panel), or stained with instant blue (bottom panel) as a loading control. (**B**) Immunoblot analysis of whole-cell lysate from parental, cKD-ABCB7L-HA, and cKD-ISU1-HA, grown in the presence or absence of ATc for 3 days and treated with puromycin for 15 minutes. Samples were separated by SDS-PAGE and labeled as in panel A. (**C**) Quantification of immunoblots in panel B. Each point represents a replicate, normalized to an instant blue signal, and no ATc set at 100%. Error bars are mean ± SD, and a one-sample *t*-test was used to compare levels in plus ATc to no ATc, *n* = 5. (**D**) Immunofluorescence assay analysis of parental and cKD-ABCB7L-HA parasites, grown in the presence of ATc for 3 days, labeled with anti-puromycin (yellow), anti-GAP45 to outline the parasites (magenta). The scale bar is 5 µm.

Given the observed decrease of many proteins involved in translation in our LFQ data (Fig. 5G), we tested whether the overall cytosolic translation rate was affected in the *Tg*ABCB7L mutant using puromycin incorporation assays. Puromycin is an amino-nucleoside antibiotic that blocks protein translation by incorporating itself into the nascent polypeptide chain during translation and blocking further synthesis. By measuring the puromycin incorporation, through immunoblot assays, the rate of protein synthesis can be measured (56). We first exposed freshly egressed parasites with either 100 µg/mL puromycin for 15 minutes or 100 µg/mL cycloheximide (a translation inhibitor), followed by 15 minutes with puromycin; and no treatment. We then looked at these parasites by immunoblot analysis with antibodies against puromycin. Substantial labeling had occurred in the puromycin-incubated parasites, while none was seen in the untreated control, showing the specificity of the assay (Fig. 7A). Parasites incubated with cycloheximide prior to puromycin labeling showed much decreased labeling, showing that the assay can detect changes in translation. Next, to assess the rate of protein translation upon *Tg*ABCB7L depletion, we grew parental and cKD-ABCB7L-HA for three days in the presence or absence of ATc, followed by puromycin labeling and immunoblot analysis. As a control, we included cKD-ISU1-HA parasites, which have a severe defect in the mitochondrial ISC pathway, but for which similar LFQ proteomic analysis suggests no decrease in translation-related components (25). Parasites depleted in *Tg*ABCB7L showed a ~40% decrease in puromycin incorporation (Fig. 7B and C), while parental or cKD-ISU1-HA parasites grown in ATc showed no decrease, suggesting that translation is decreased only when the CIA pathway is disrupted. Depletion of ISU1 results in a growth phenotype of similar severity to *Tg*ABCB7L; thus, the decrease in translation defect is not a general phenotype of disrupted Fe-S cluster assembly, but rather a specific defect of *Tg*ABCB7L depletion. A similar decrease in puromycin incorporation was seen in cKD-ABCB7L-HA, but not parental, cells grown for three days in ATc in an immunofluorescence-based version of the assay (Fig. 7D).

The decrease in abundance of proteins involved in translation and decrease in puromycin incorporation upon *Tg*ABCB7L depletion suggests that disruption of the CIA pathway results in a moderate defect in the translation rate in the cell, which may be due to a decrease in abundance of translational components (Fig. 5G), including potentially Fe-S cluster containing components of the translation machinery, such as ABCE1 and DPH2 (Table 1).

Overall, our study provides evidence that the ABCB7-like protein *Tg*ABCB7L, encoded by TGGT1_269000, performs a role similar to ABCB7 homologs in other model systems. We show that *Tg*ABCB7L is required for the biogenesis of cytosolic and nuclear Fe-S proteins, as well as protein translation, suggesting a role in cytosolic Fe-S biosynthesis.

## DISCUSSION

Fe-S clusters are ubiquitous and essential inorganic cofactors for numerous essential life processes that have dedicated biosynthetic pathways. Despite some variation in different lineages, the core machinery of these biosynthetic pathways is remarkably conserved. Recently, studies on Fe-S cluster biosynthesis in the important eukaryotic pathogen *Toxoplasma gondii* have revealed the presence and functional importance of components from the ISC pathway in the mitochondria (25, 27), the SUF pathway in the apicoplast (25, 26), and the CIA pathway in the cytosol (27). Understanding their role in *Toxoplasma*, and uncovering any lineage-specific features, is therefore important in the effort to combat these pathogens. These studies have also described some lineage-specific aspects of otherwise conserved components. For example, the cytosolic scaffold NBP35, which plays a central role in assembling cytosolic clusters, was found to be bound to the outer surface of the mitochondria (27), whereas in other model systems, it is a soluble cytosolic protein, with one study suggesting a nuclear localization (57). The exact reason for NBP35’s re-localization remains unclear, but it may highlight the connection between the CIA and ISC pathways in apicomplexan parasites and reflect the complex evolutionary history of these proteins in eukaryotes (58–60).

To test explicitly for a connection of the mitochondrial and cytosolic Fe-S assembly pathways in *Toxoplasma*, we sought evidence of a transporter equivalent to known ABCB7 that transports mitochondrial Fe-S intermediates from the ISC to CIA pathways. Homology searches by BLAST highlighted TGGT1_269000, also identified in reference 25. However, this gene was also suggested as a putative homolog of the closely related ABCB6 (31). Studies in mammals proposed ABCB6 as a mitochondrial outer membrane transporter involved in porphyrin transport (31), although subsequent studies question this localization and role (11). Our phylogenetic, and subsequent functional, analyses provide evidence that TGGT1_269000 is not a *Toxoplasma* ABCB6 ortholog. First, a second *Toxoplasma* protein, TGGT1_273360, is a more likely ABCB6 candidate, forming a well-supported clade that includes characterized ABCB6 homologs (Fig. 1B; Fig. S2). TGGT1_273360 has a predicted endomembrane vesicle and Golgi localization (33), consistent with a subsequent study which demonstrated a Golgi localization of the mammalian ABCB6 (61). In addition, TGGT1_273360 is predicted to be dispensable for *Toxoplasma* tachyzoite fitness (40), which is at odds with the severe fitness consequences seen in mutants of ABCB7 homologs in other model systems. Second, our functional analysis did not detect any defect in heme-containing proteins. Based on previous studies, it should be apparent in our analysis if processes involving heme proteins were affected. Mutants in the heme biosynthetic protein CPOX displayed a severe defect in mitochondrial oxygen consumption (62), whereas our mutant had no observable defect in mOCR (Fig. 4FI). Another study (54) looking at the heme biosynthetic protein UROD showed a decrease in mOCR and ECAR as well as a decrease in the abundance of the cytochrome *c -*A and -B proteins. Cyt *c*-B was detected in our LFQ proteomic data set but was not decreased in abundance. Overall, we did not find phenotypes consistent with a defect in heme protein biogenesis. By contrast, we found phenotypes consistent with functional equivalence with ABCB7.

Our phylogenetic analysis of ABCB6 and 7 orthologs across eukaryotes showed that TGGT1_269000 is part of a single well-resolved clade of myzozoa. This group does not clearly resolve with either group of studied ABCB6 or ABCB7 homologs, meaning we could not unambiguously assign TGGT1_269000 as the *Toxoplasma* ortholog of ABCB7 based on sequence alone. However, based on our functional studies which show similar phenotypes to mutants in yeast, human, and plant ATM1/ABCB7 and ATM3, TGGT1_269000 seems to have the same function as ABCB7. This role is likely conserved across the myzozoan proteins found in the same orthogroup. Notably, a TGGT1_269000 ortholog is present in *Cryptosporidium*, which has highly functionally reduced mitochondria (mitosomes) that no longer contain an mETC, but are thought to retain a role in Fe-S cluster biogenesis (3). Therefore, the retention of TGGT1_269000 in this species provides additional evidence that it plays a role connected to the Fe-S cluster biogenesis pathway.

Similar to *Toxoplasma*, the genomes of *Plasmodium falciparum* also contain numerous proteins with sequence similarity to ABCB6 and 7. One of these, PF3D7_1447900, named MDR2, has previously been suggested as a functional homolog of ABCB7 (63), but its dispensability for blood-stage parasites (64) raised questions if it truly performed this usually essential role (65). In our phylogenetic analysis, PF3D7_1447900 appears in the well-resolved group of ABCB6 homologs, including *Toxoplasma* TGGT1_273360, suggesting this is a *Plasmodium* ABCB6 ortholog. Our analysis suggests that PF3D7_1352100 is the most likely candidate for a *Plasmodium* protein performing a similar function to ABCB7, as it groups within the myzozoan clade which contains TGGT1_269000. PF3D7_1352100 is predicted to be essential based on a genome-wide *piggyBac* mutagenesis screen (66), which would appear consistent with a conserved and essential role in connecting the mitochondrial and cytosolic Fe-S biogenesis pathways. In addition, a recent study of the (NIA)_2_ complex member, mACP, in *Plasmodium* suggested that mETC dysfunction only partially explained the death phenotype and that mACP may also be required for cytosolic Fe-S biogenesis, suggesting that the *Plasmodium* homolog of ABCB7 would likely be essential (65). Ultimately, future functional studies will be needed to confirm if PF3D7_1352100 is the functional equivalent of ABCB7 in *Plasmodium*.

Studies in a wide array of organisms clearly show that the role of the ABCB7 transporter in connecting the mitochondrial ISC and cytosolic CIA pathways is conserved (3). Downstream cytosolic Fe-S proteins are dependent on the cysteine desulfurase activity of the mitochondrial NFS1 enzymes, and the resulting sulfur intermediate that is transported out of the mitochondrial matrix by ABCB7. ABCB7 mutants in yeast and mammals are impaired in thio-modification of cytosolic tRNAs, protein translation, DNA replication, and genome maintenance (3, 22, 55, 67, 68). This is consistent with what we find in our study, with LFQ proteomic data pointing to significant defects in translation and DNA metabolism, as well as Fe-S proteins involved in these processes (Fig. 5F through H; Table 1). Experiments also confirmed the specific depletion of the ribosome splitting factor ABCE1 and the DNA polymerase subunit POLD1. Further studies could test whether thio-modification of tRNAs is affected. Growth assays (Fig. 3D) and ECAR measurements from extracellular flux analysis (Fig. 4F) show that parasites are still viable when these assays are performed and so act as a useful control that observed defects are specific and not due to a general decrease in parasite viability. Other studies of ABCB7 homologs, for example in plants, have assayed cytosolic Fe-S cluster defects through in-gel activity assays of enzymes containing Fe-S clusters such as aldehyde oxidase, xanthine dehydrogenase, and cytosolic isoforms of aconitase (14, 69–71). *Toxoplasma* lacks obvious homologs of these enzymes or does not have clear cytosolic-only isoforms and so we were unable to a perform similar analysis here.

We previously predicted *T. gondii* to have 64 Fe-S cluster containing proteins (25) using the computational tool MetalPredator (48). However, predicting the ability of proteins to bind Fe-S clusters from sequence alone has long proved challenging (72) and other species contain many more Fe-S proteins. For example, a recent inventory of *E. coli* Fe-S proteins numbered 144 (72). In eukaryotes, up to 1% of the proteome is predicted to contain Fe-S clusters (73) (which would be 84 proteins if this trend is observed in *Toxoplasma*). Given these facts, it is plausible that *Toxoplasma* contains more, as yet unidentified, Fe-S proteins. While many Fe-S proteins are degraded if they do not bind to a cluster (51), not all proteins are destabilized. Our previous study using LFQ proteomics to study a mutant of the mitochondrial Fe-S cluster synthesis pathway highlighted a sharp decrease in abundance for Fe-S proteins of the mETC, SDHB, ApiCox13, and Rieske (25). While *Tg*ABCB7L localizes to the mitochondrion, LFQ analysis of the corresponding mutant highlighted a completely different profile, as the only MetalPredator-predicted candidates found to be less abundant were cytosolic or nuclear. The data both suggest that the changed proteins are bona fide Fe-S proteins and confirm the involvement of *Tg*ABCB7L in the CIA pathway. Although LFQ is a useful approach for the validation of clients that are highly responsive to lack of Fe-S cluster occupancy, as not all Fe-S proteins become unstable in the absence of the cofactor, more specialized techniques like chemoproteomics approaches will be required to uncover new Fe-S client proteins (72).

Our previous study on mitochondrial Fe-S biosynthesis found a major mutant phenotype to be the initiation of stage conversion into bradyzoites (25). This phenotype was also seen in other mitochondrial mutants, for example of the mETC or mitochondrial translation (25, 39). This phenotype was also seen in the *Tg*ABCB7L mutant. The integration of environmental or metabolic cues that lead to conversion into bradyzoites is not fully understood. Stage conversion in the *Tg*ABCB7L mutant may be triggered by metabolic signals derived from sensing a lack of functional Fe-S clusters in important enzymes. Another possibility, given the decreased abundance of many proteins involved in translation in the *Tg*ABCB7L mutant, is that a block in translation could induce the parasite to begin differentiation. Previous studies have shown translational control to be an important aspect of initiating differentiation (74–78). More work needs to be done to understand how stress and metabolic deficiencies influence a parasite’s decision to initiate conversion.

A link between the ISC and CIA pathways, mediated by a mitochondria ABCB transporter, has been observed in a wide variety of model systems. However, there are exceptions to be found in microbial parasites, especially those containing reduced mitochondria (mitochondria-related organelles—MROs), which apparently lack ABCB7 homologs. The diplomonad intestinal parasite *Giardia*, which contains homologs of both CIA and ISC pathway components, lacks an ABCB7 homolog (79, 80), suggesting an alternative export route for the NFS1-generated sulfur intermediate (3) or raising the possibility that the two pathways are unconnected in this species. The anaerobic protist *Pygsuia biforma* lacks an ABCB7 homolog as well as ISC pathway components (81). Two amoebazoans, *Entamoeba histolytica* and *Mastigamoeba balamuthi*, lack ABCB7 homologs, as well as ISC components, instead utilizing the bacterial-like NIF pathway (3). A more extreme example is the Oxymonad *Monocercomonoides exilis* which lacks a mitochondrion, and instead supplies its CIA pathway by a cytosolic SUF pathway (82, 83). These examples serve to highlight the biochemical diversity of non-canonical Fe-S assembly pathways present in the microbial parasites and highlight the utility of studying these pathways to gain a full picture of Fe-S assembly in eukaryotes. While our data clearly demonstrate a conserved, canonical function of *Tg*ABCB7L in cytosolic Fe-S assembly, *Toxoplasma* may also possess unusual features in its Fe-S assembly pathways that remain to be discovered and could be exploited for potential therapeutic approaches.

## MATERIALS AND METHODS

### Database sampling and phylogenetic analysis

A custom database was collated that included data for relevant eukaryote species from UniProt (84) and the Marine Microbial Eukaryote Transcriptome Sequencing Project (85), as well as data for apicomplexans and close relatives generated by two previous studies (86, 87). This custom database was then sampled for ABCB6 and ABCB7 homologs using the program blastp (BLAST+ version 2.11.0 [88]) for which characterized ABCB6 and ABCB7 homologs from selected model organisms were used as search queries. The data set of possible ABCB6 and ABCB7 homologs obtained from these searches was then clustered with CD-HIT (89) to remove highly similar sequences, thereby reducing the overall redundancy of the data set. Iterative rounds of alignment using mafft (MAFFT version 7.475 [90]), conserved site selection using trimal with “gappyout” mode (trimAl version 1.4 [91]), tree inference with FastTreeMP (FastTree version 2.1.11 (92) using the default settings, and manual removal of sequences were then performed to further reduce data set redundancy, as well as to identify and remove sequences that aligned poorly or were so dissimilar from the majority of the sequences in the data set that they were unlikely to be ABCB6 or ABCB7 homologs, but rather false positives of the described sampling strategy. The final curated data set that these methods obtained was then aligned using mafft-linsi (MAFFT version 7.475 [90]) and conserved sites were selected using trimal with “gappyout” mode (trimAl version 1.4 [91]). A phylogeny was then inferred from this data set using the program iqtree2 (IQ-TREE version 2.1.2 [93]) with 1,000 ultrafast bootstrap replicates (UFBoot2 [8]) and using the best-fitting model, LG + F + I + G4 (94, 95), that was chosen according to the Bayesian Information Criterion by ModelFinder (96), all implemented within iqtree2. The protein sequences, alignment, and tree inference output files used to generate the phylogeny are provided at https://doi.org/10.6084/m9.figshare.26840203.

### Cell culture

*Toxoplasma gondii* tachyzoites were cultured in human foreskin fibroblasts (HFF), sourced from ATCC (SCRC-1041). HFFs and parasites were cultured in Dulbecco’s modified Eagle’s medium (DMEM), containing 4.5 gL^−1^ glucose, supplemented with 10% (vol/vol) fetal bovine serum, 4 mM L-glutamine and penicillin/streptomycin and gentamycin antibiotics and grown at 37°C with 5% CO_2_. When needed, anhydrotetracycline (ATc) was added to the medium at a final concertation of 0.5 µM.

### Parasite genetic manipulation

Parasite genetic manipulation was performed as described previously (35, 39). CRISPR-guided promoter replacement and C-terminal triple HA epitope tagging were performed in the TatiΔ*ku80* background (32). gRNAs targeting the start or stop codon of the GOI were designed using the ChopChop tool (https://chopchop.cbu.uib.no/) and cloned into a vector containing the U6 promoter and expressing CAS9-GFP (Tub-Cas9-YFP-pU6-ccdB-tracrRNA) (97) using the BsaI restriction site. For promoter replacement, the pDTS4myc plasmid (32) was used as a template for amplification of DHFR selectable cassette and ATc repressible promoter by PCR. For HA epitope tagging, the CAT selection and triple HA epitope tag were amplified by PCR from p3HA.LIC.CATΔpac plasmid (32, 98). The gRNA/CAS9 vector-PCR product mixture was transfected into the relevant parental line by electroporation and cassette integration selected by antibiotics. Positive clones were isolated by serial dilution and confirmed by PCR analysis, using the primers from Table S4.

Downregulation of transcript in the promoter replacement line was confirmed by qRT-PCR as described previously (35, 39), using actin (TGGT1_209030) as an internal control.

For the complementation of the cKD-ABCB7L-HA cell line, the *Tg*ABCB7L sequence with added MfeI and NsiI restriction sites was synthesized (GenScript) and cloned into a pTUB8mycGFPMyoATy expression vector (42). The vectors were electroporated into cKD-ABCB7-HA and integration was selected with mycophenolic acid (25 mg mL^−1^) and xanthine (50 mg mL^−1^).

### Growth analysis

Plaque assay: HFF monolayers were infected with freshly egressed tachyzoites and grown in the presence or absence of ATc for eight days. Cells were fixed with methanol and stained with a 0.4% crystal violet solution. 50 plaques per condition were measured from three biological replicates using Image J.

Replication assay: parental and cKD-ABCB7L-HA parasites were grown in the presence or absence of ATc for two days and then allowed to infect a fresh HFF monolayer for another day in the same conditions. Immunofluorescence assays were performed as described below using the GAP45 antibody (99). The number of vacuoles containing one, two, four, or eight and more parasites were counted for over 100 vacuoles per line per experiment. Three independent experiments were performed.

### SDS and native PAGE and immunoblot analysis

SDS-PAGE analysis was performed as described previously (35). Immunoblot analysis after SDS-PAGE was performed with relevant primary antibodies: anti-HA (1:500, anti-rat, Sigma), anti-TOM40 (1:2,000, anti-rabbit [38]), anti-Ty (1:1,000, anti-mouse [100]), anti-ATPβ (1:2,000, anti-rabbit, Agrisera AS05 085), and anti-CDPK1 (1:10,000, anti-guinea pig [101]). Immunolabeling was quantified using FIJI software, and each HA sample was normalized to the TOM40 loading control.

For BN-PAGE, parasites were resuspended in solubilization buffer (750 mM aminocaproic acid, 50 mM Bis-Tris-HCl [pH 7.0], 0.5 mM EDTA, 1% [wt/vol] βDDM or SDS) and incubated on ice for 30 minutes. The samples were then centrifuged at 16,000 × *g* at 4°C for 30 minutes. For βDDM-solubilized samples, the supernatant was combined with sample buffer containing Coomassie G250, resulting in a final concentration of 0.25% DDM and 0.0625% Coomassie G250. For SDS-solubilized samples, the β-mercaptoethanol was added and the sample was heated at 95°C for five minutes before adding sample buffer containing G250. Samples were separated on a native PAGE 4-16% Bis-Tris gel and transferred to PVDF membrane (0.45 µm, Hybond) using wet transfer in Towbin buffer (25 mM Tris, 192 mM glycine, 10% methanol) for 60 minutes at 100 V, before immunoblot analysis. Bovine mitochondrial membranes were used as a molecular weight marker.

### Immunofluorescence assay

To assess localization, immunofluorescence assays were performed as described previously (35) using anti-HA (1:500, anti-rat, Sigma), anti-MYS (1:1,000, anti-rabbit [36]), anti-Ty (1:800, anti-mouse [100), and anti-GAP45 (1:1,000, anti-rabbit [99]) primary antibodies. Images were acquired as Z-stacks on a DeltaVision Core microscope (Applied Precision) using the 100× objective and images were processed and deconvolved using the SoftWoRx and FIJI software.

To assess mitochondrial morphology, parasites were grown in an HFF monolayer on a glass coverslip in the presence or absence of ATc for three days and an immunofluorescence assay was performed using primary antibodies against the mitochondrial protein TOM40 (1:1,000, anti-rabbit [38]). Mitochondrial morphology for each vacuole was scored as “normal” or “abnormal” based on the presence of distinctive lasso-shaped mitochondrial morphology (36). Four independent experiments were performed and over 150 vacuoles were scored for each replicate.

### Respiratory measurements

Extracellular flux analysis: basal oxygen consumption rate (OCR) and extracellular acidification rate (ECAR) were measured using a Seahorse XF HS Mini Analyser (Agilent Technologies) as described previously (39).

### Puromycin incorporation assay

The puromycin incorporation assay to assess protein translation was adapted from reference 49. Parasites were grown in the presence or absence of ATc for three days. Freshly egressed parasites were then filtered through a 3.0-µm polycarbonate filter to remove host cell debris and washed once with media. Parasites were then incubated with media containing 10 µg mL^−1^ puromycin (puromycin dihydrochloride from *Streptomyces alboniger*, Sigma) for 15 minutes at 37°C, before washing with ice-cold PBS. As a control, parasites were incubated with 100 µg mL^−1^ cycloheximide for 10 minutes prior to puromycin treatment. Whole-cell samples were then analyzed by immunoblot using an anti-puromycin antibody (1:2,000, anti-mouse, clone 12D10, Merck). As a loading control, a duplicate SDS-PAGE was performed, and gels were incubated in InstantBlue Coomassie protein stain (abcam) for 1 hour to visualize total protein. Puromycin labeling was quantified using Odyssey Image Studio (Version 5.2) software. Total protein was quantified by densitometry analysis using FIJI software. For the immunofluorescence-based assay, parasites were grown in an HFF monolayer on a glass coverslip in the presence of ATc for three days and then incubated with media containing 10 µg mL^−1^ puromycin for 10 minutes at 37°C. Cells were then fixed and the immunofluorescence assay carried out as described above with an anti-puromycin antibody at a concentration of 1:1,000.

### Immunoprecipitation

For immunoprecipitation of cKD-ABCB7L-HA/ABCB7L-Ty, 1.5 × 10^8^ cells were divided into three equal aliquots and incubated in lysis buffer (1% digitonin, 50 mM Tris-HCL [pH 7.4], 150 mM NaCl, 2 mM EDTA) for 10 minutes on ice and then 30 minutes on a rotator at 4°C. One aliquot was retained as an input sample while the other two were cleared by centrifugation at 18,000 × *g* at 4°C for 30 minutes and the supernatant was incubated with either anti-HA or anti-Ty agarose beads overnight at 4°C. The agarose beads were pelleted, and the supernatant was TCA precipitated to give the “unbound” fraction. The beads were then washed in lysis buffer with 0.1% digitonin and resuspended in laemmli buffer to give the “bound” fraction. All fractions were then subjected to immunoblot analysis with antibodies against HA, Ty, TOM40, and CDPK1, as above.

### Sodium carbonate extractions

Sodium carbonate extractions were performed as described previously (39). Briefly, parasites were treated with 100 mM Na_2_CO_3_, pH 11.5, and incubated at 4°C for two hours. The pellet, containing integral membrane proteins, and supernatant, containing peripheral membrane and non-membrane associated proteins, were separated by ultracentrifugation at 189,000 × *g*. To test the solubility of proteins, parasites were resuspended in 1% Triton X-100, incubated at 4°C for two hours, and pellet and supernatant fractions were separated by centrifugation at 16,000 × *g*. Fractions were then tested by immunoblot analysis.

### Cyst quantification

For cyst labeling, intracellular parasites grown in the presence of ATc were processed as above for IFA. Samples were incubated with a biotinylated version of the lectin from *Dolichos biflorus* (Sigma Aldrich, #L6533-5MG) at 1:300, followed by incubation with a streptavidin-fluorescein isothiocyanate (FITC) conjugate (ThermoFisher #SA10002) at 1:500. Parasites were co-stained with the rabbit anti-IMC3 antibody (102) at 1:1,000. At least 100 cysts/vacuoles were counted for each condition in randomly selected fields under a Zeiss AXIO Imager Z2 epifluorescence microscope of the Montpellier Ressources Imagerie platform, driven by the ZEN software v2.3 (Zeiss).

### Quantitative label-free mass spectrometry

The analysis was performed as described previously (25). Parasites of the TATiΔ*ku80* and cKD-ABCB7L-HA cell lines were grown for three days in the presence of ATc before being mechanically released from their host cells, filtered on a glass wool fiber column, and washed in Hanks’ balanced salt solution (Gibco). After parasite pellet resuspension in SDS lysis buffer (50 mm Tris-HCl [pH 8], 10 mm EDTA [pH 8], 1% SDS), protein quantification was performed with a bicinchoninic acid assay kit (Abcam) and for each condition, 20 µg of total proteins was separated on a 12% SDS-PAGE run for 20 minutes at 100 V, stained with colloidal blue (Thermo Fisher Scientific). Each lane was cut in three identical fractions and trypsin digestion and peptide extraction were performed as described previously (103). The LC-MS/MS experiments were performed with an Ultimate 3000 RSLC nano system (Thermo Fisher Scientific Inc., Waltham, MA, USA) interfaced online with a nano easy ion source and an Exploris 240 Plus Orbitrap mass spectrometer (Thermo Fisher Scientific Inc., Waltham, MA, USA).

The .raw files were analyzed with MaxQuant version 2.0.3.0 using default settings (104). The minimal peptide length was set to 6. Up to two missed cleavages were allowed. The mass tolerance for the precursor was 20 and 4.5 ppm for the first and the main searches, respectively, and for the fragment ions was 20 ppm. The files were searched against *T. gondii* proteome (March 2020; https://www.uniprot.org/proteomes/UP000005641; 8,450 entries). Identified proteins were filtered according to the following criteria: at least two different trypsin peptides with at least one unique peptide, an *E* value below 0.01 and a protein *E* value smaller than 0.01 were required. Using the above criteria, the rate of false peptide sequence assignment and false protein identification was lower than 1%. Proteins were quantified by label-free method with MaxQuant software using unique and razor peptide intensities (105). Statistical analyses were carried out using RStudio package software. The protein intensity ratio (protein intensity in mutant/protein intensity in parent) and statistical tests were applied to identify the significant differences in the protein abundance. Hits were retained if they were quantified in at least three of the four replicates in at least one experiment. Proteins with a significant (*P* < 0.05 with Benjamini correction) change in a quantitative ratio were considered as significantly upregulated and downregulated, respectively. Additional candidates that consistently showed the absence or presence of LFQ values versus the control in at least three out of the four biological replicates were also considered.

## Data Availability

All raw MS data and MaxQuant files generated have been deposited to the ProteomeXchange Consortium via the PRIDE partner repository (https://www.ebi.ac.uk/pride/archive) with the data set identifier PXD048386. The protein sequence, alignment, and tree inference output files used to generate the phylogeny in Fig. 1B and Fig. S2 are provided at https://doi.org/10.6084/m9.figshare.26840203. .

## SUPPLEMENTAL MATERIAL

Supplemental Material mbio.00872-24-s0001.docx Fig. S1-S6.

Table S1 mbio.00872-24-s0002.xlsx Summary of label-free quantitative proteomics results.

Table S2 mbio.00872-24-s0003.xlsx Proteins of increased or decreased abundance upon depletion of ABCB7L.

Table S3 mbio.00872-24-s0004.xlsx Localization analysis of proteins of increased or decreased abundance upon depletion of ABCB7L.

Table S4 mbio.00872-24-s0005.xlsx Summary of oligonucleotides used in this study.

ASM does not own the copyrights to Supplemental Material that may be linked to, or accessed through, an article. The authors have granted ASM a non-exclusive, world-wide license to publish the Supplemental Material files. Please contact the corresponding author directly for reuse.
