# Supplementary material for: The Toxoplasma gondii mitochondrial transporter ABCB7L is essential for the biogenesis of cytosolic and nuclear iron-sulfur cluster proteins and cytosolic translation
Source: mBio. 2024 Aug 29;15(10):e00872-24. doi: 10.1128/mbio.00872-24 (PMC11481526; doi:10.1128/mbio.00872-24)
Supplement: Supplemental Material — Fig. S1-S6. [file mbio.00872-24-s0001.docx]

## Supplemental Figures


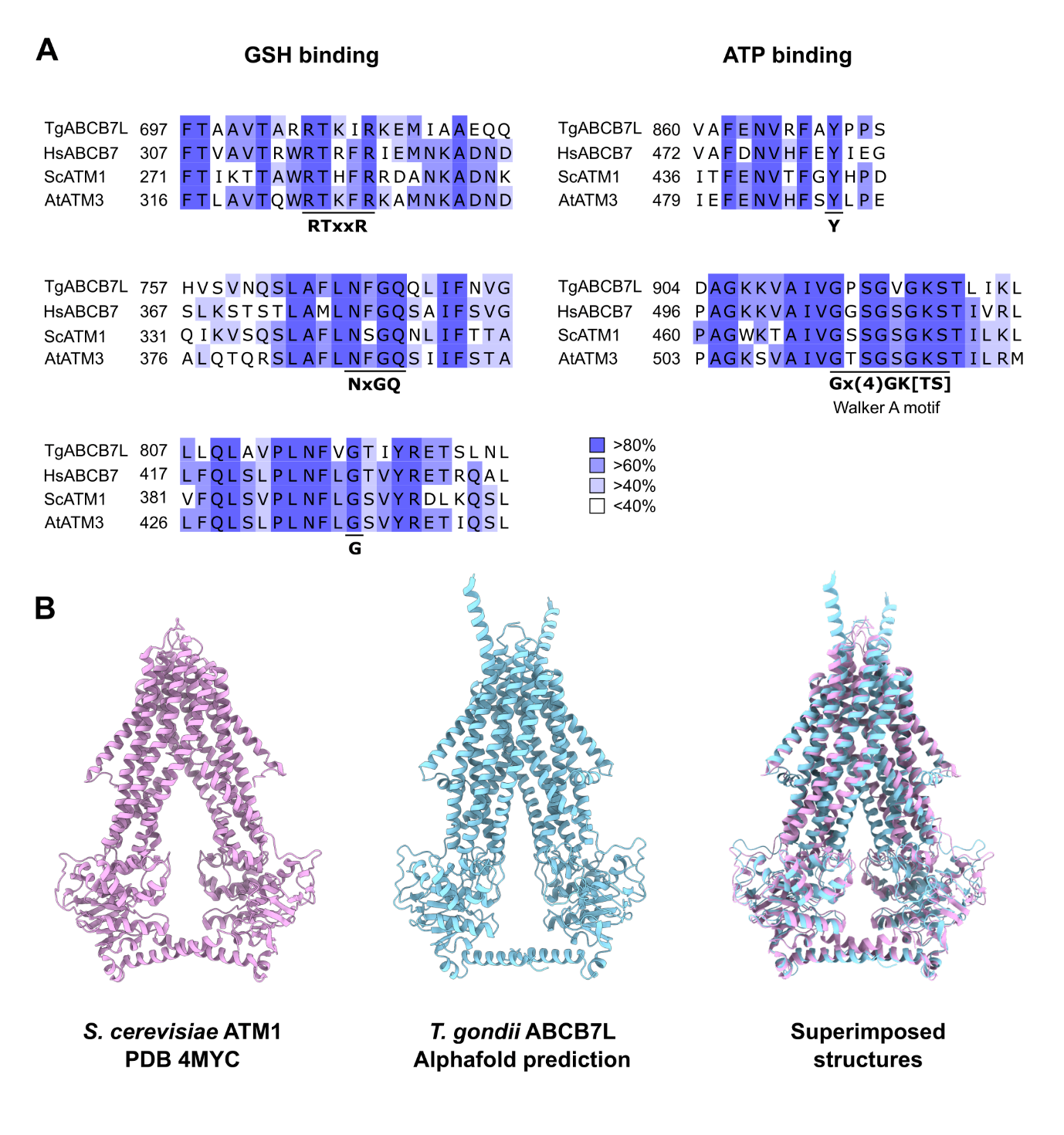


**Figure S1 Alignments and structural comparison of ABCB7 homologs.**

(A) Alignments of TGGT1_269000, HsABCB7 (O75027), ScATM1 (P40416) and AtATM3 (Q9LVM1), showing conservation of key residues involved in glutathione and ATP binding. Alignments were made using Clustal Omega and visualized using JalView. Colour coding depicts percent identity. (B) ATM1 dimer structure (left) from *Saccharomyces cerevisiae* (PDB 4YMC, (1)) (left), predicted structure of *Toxoplasma gondii* TGGT1_269000 (Alphafold2 (2, 3)) (centre) and superimposition of the two structures (RMSD = 1.264 Å, TM-align score of 0.88, where scores above 0.5 indicate similarity (4)) (right). Unstructured loop regions of ABCB7L have been truncated for clarity. Visualized using ChimeraX (5).


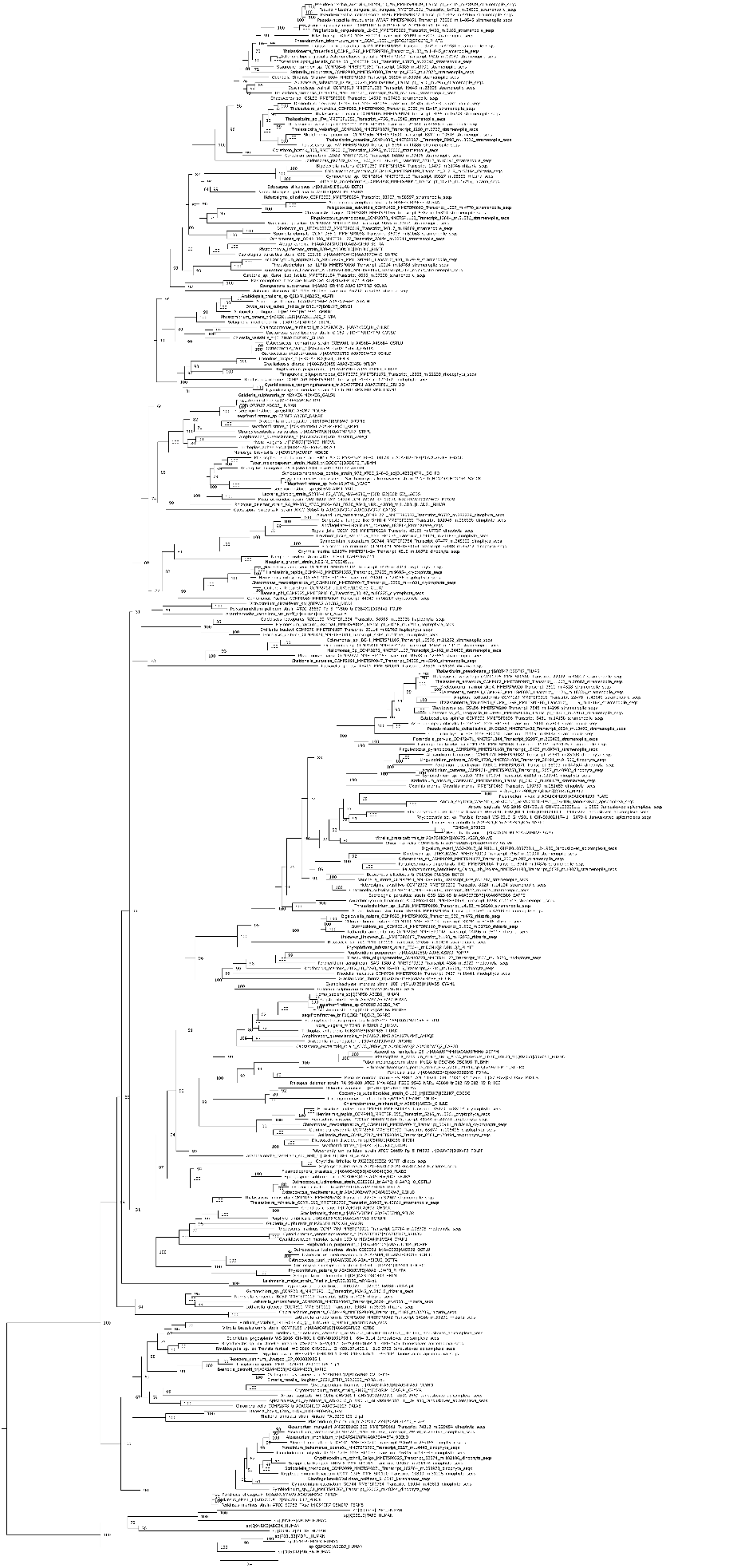


**Figure S2. Phylogeny of ABCB6 and 7 homologues from various eukaryotes**

A fully expanded version of the phylogenetic tree shown in figure 1B, with no collapsed clades and displaying sequence accessions. The protein sequences, alignment, and tree inference output files used to generate the phylogeny are provided in Supplementary Data 1.


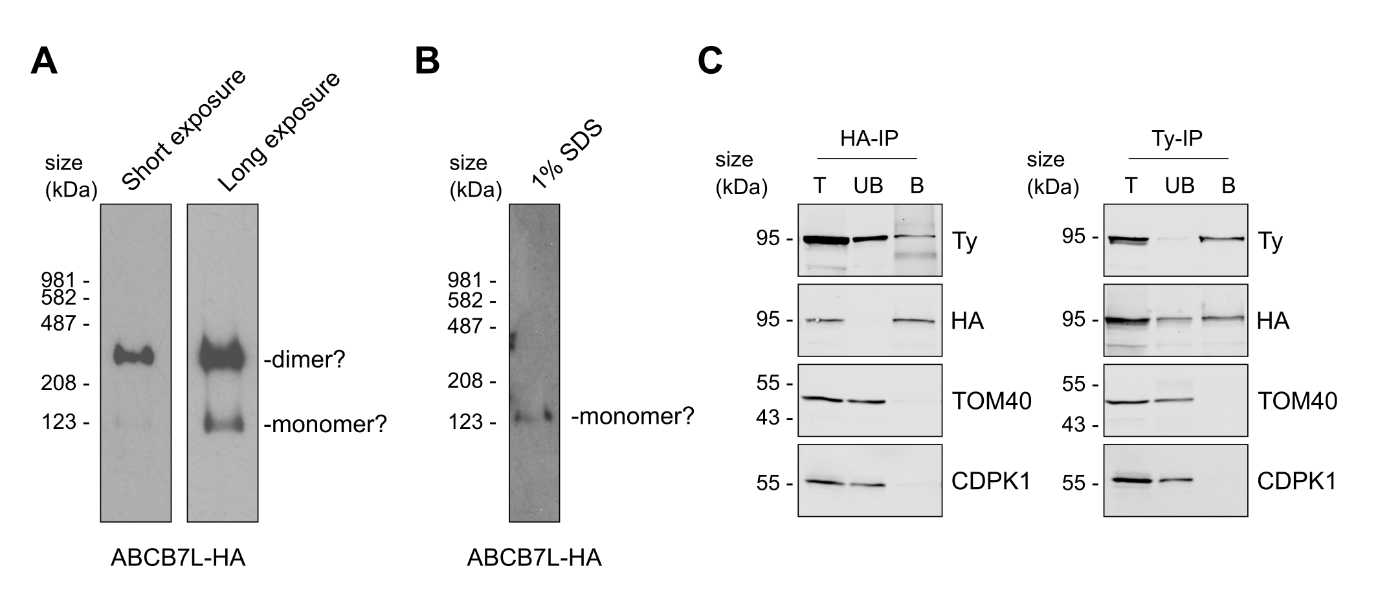


**Figure S3 ABCB7L homodimer formation.**

(A) BN-PAGE analysis of ABCB7L-HA parasites extracted in 1% βDDM, immunolabelled with anti-HA. Two exposure lengths shown, and putative monomer and dimer bands indicated. The short exposure is also used in Fig. 1C (B) BN-PAGE analysis of ABCB7L-HA parasites extracted in 1% SDS, immunolabelled with anti-HA. (C) Immunoblot analysis of whole cell lysate extracted from cKD-ABCB7L-HA + ABCB7L-Ty and immunoprecipitated with anti-HA or anti- Ty antibody coupled beads, to produce total lysate (T), unbound (UB) and bound (B) fractions. Samples were separated by SDS-PAGE, blotted, and detected using anti-HA and anti-Ty antibodies to label immunoprecipitated proteins, and anti-TOM40 as an unrelated mitochondrial protein control and anti-CDPK1 as an unrelated protein control.


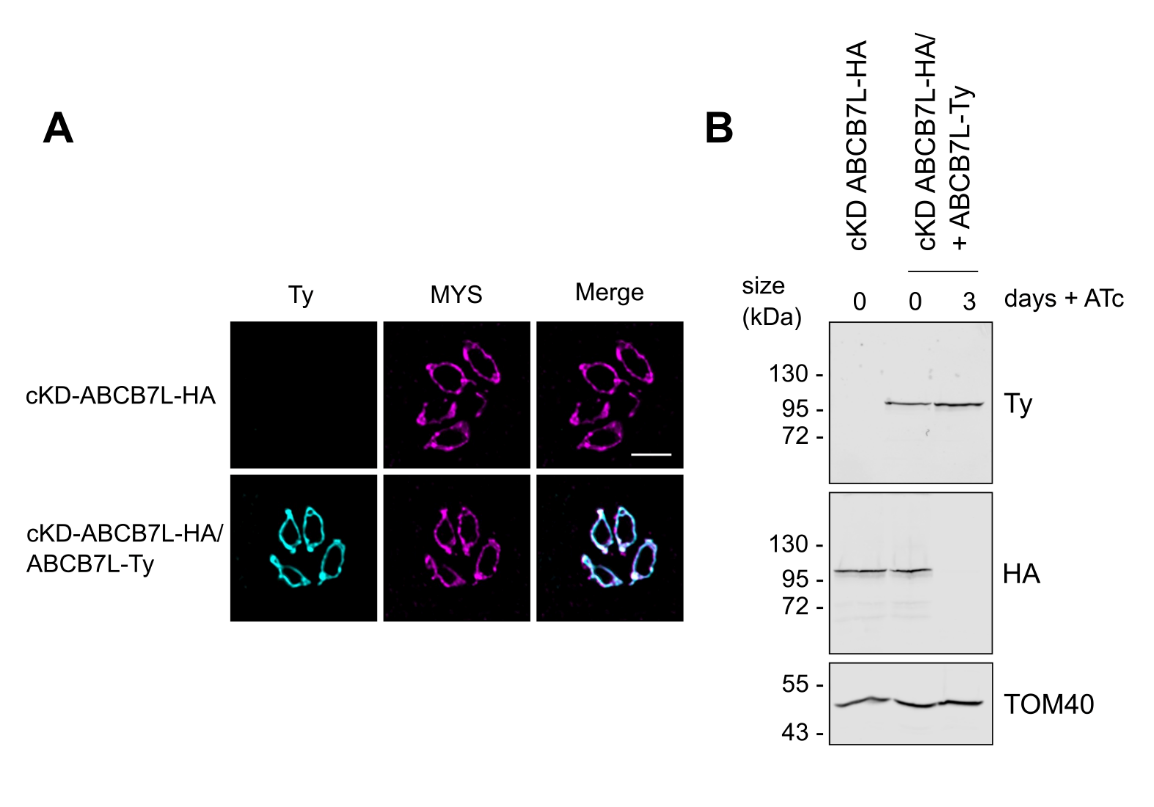


**Figure S4 Validation of the complementation of cKD-ABCB7L-HA.**

(A) Immunofluorescence assay analysis of cKD-ABCB7L-HA and cKD-ABCB7L-HA + ABCB7L-Ty parasites, labelled with anti-Ty to detect ABCB7L-Ty (cyan), and the mitochondrial marker protein MYS (magenta). Scale bar is 5 µM. (B) Immunoblot analysis of whole cell lysate extracted from cKD-ABCB7L-HA and cKD-ABCB7L-HA + ABCB7L-Ty parasites treated with ATc for zero or three days. Samples were separated by SDS-PAGE, blotted, and detected using anti-Ty, to visualize ABCB7L-Ty, anti-HA, to visualize ABCB7L-HA, and anti-TOM40 as a loading control.


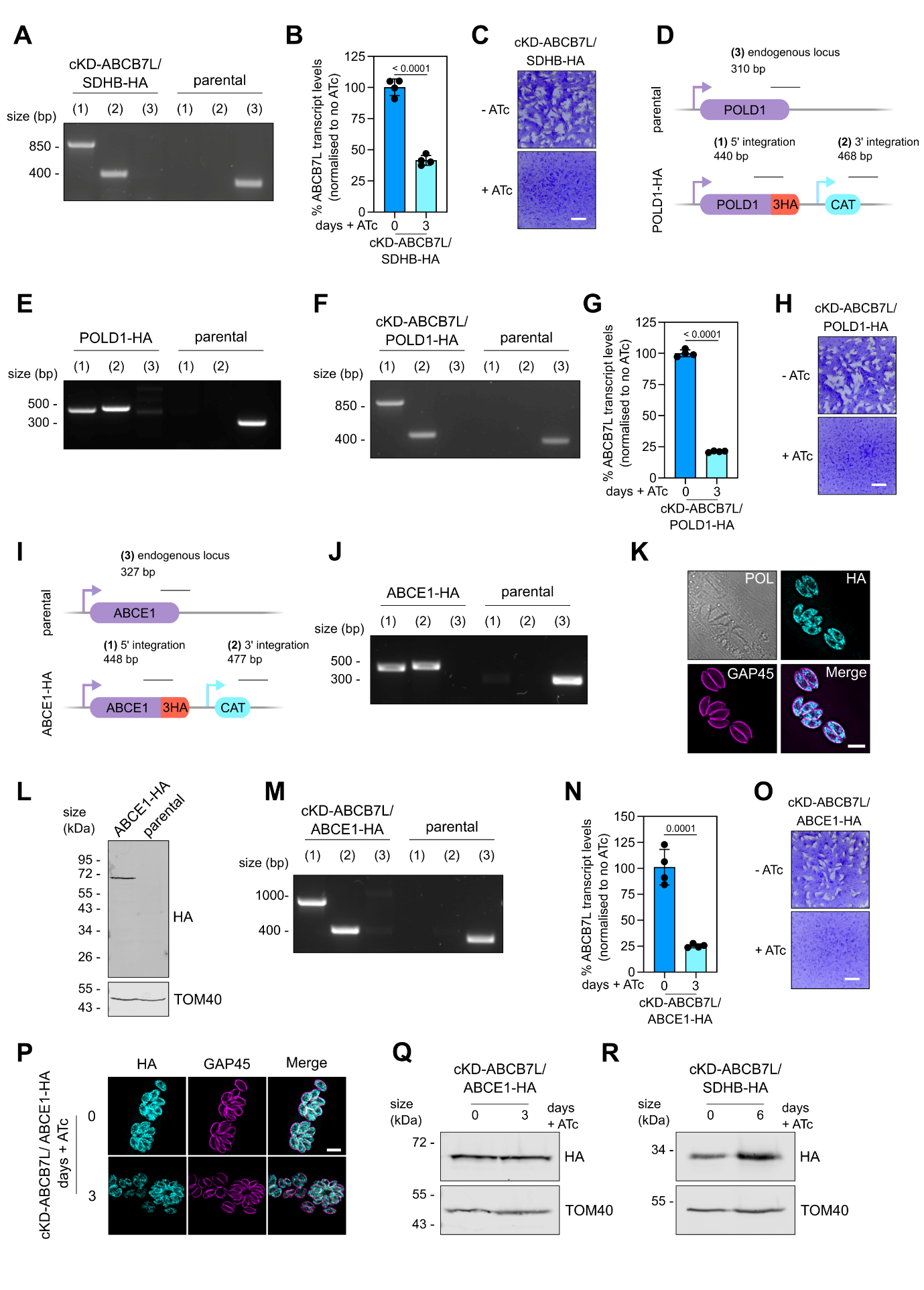


**Figure S5 Validation of the genetic manipulation performed for each of the described parasite lines.**

(A) PCR to test the integration of the DHFR selection cassette and the regulatable promoter into the endogenous locus of *Tg*ABCB7L in the SDHB-HA background, as outlined in Fig. 2A. (B) Relative transcript levels of *Tg*ABCB7L in cKD-ABCB7L/ SDHB-HA line after three days Anhydrotetracycline (ATc) treatment, measured by qRT-PCR. The mean of zero-day ATc set at 100% and error bars are mean -/+ S.D., and an unpaired t-test used to compare transcript levels in plus ATc to no ATc, n=4. (C) Plaque assay of cKD-ABCB7L/ SDHB-HA grown in the presence or absence of ATc for 8 days. Scale bar is 5 mm (D) Schematic of the strategy used to C-terminally HA-epitope tag the POLD1 protein. The expected size of integration PCRs are shown. (E) PCR to test integration of the HA-epitope tag and CAT selection cassette into the endogenous locus of POLD1, as outlined in *(D)*. (F) PCR to test integration of DHFR selection cassette and the regulatable promoter into the endogenous locus of *Tg*ABCB7L in the POLD1-HA background, as outlined in Fig. 2A. (G) qRT-PCR analysis of cKD-ABCB7L/ POLD1-HA, as in *B*. (H) Plaque assay of cKD-ABCB7L/ POLD1-HA as in *C*. (I) Schematic of the strategy used to C-terminally HA-epitope tag the ABCE1 protein. The expected size of integration PCRs are shown. (J) PCR to test the integration of the HA-epitope tag and CAT selection cassette into the endogenous locus of ABCE1, as outlined in *(I)*. (K) Immunofluorescence assay analysis of ABCE1-HA parasites, labelled with anti-HA to detect ABCE1-HA (cyan), and the GAP45 (magenta). Scale bar is 5 µM. (L) Immunoblot analysis of whole cell lysate extracted from ABCE1-HA and parental parasites. Samples were separated by SDS-PAGE, blotted, and detected using anti-HA, to visualize ABCE1-HA, and anti-TOM40 as a loading control. (M) PCR to test integration of DHFR selection cassette and the regulatable promoter into the endogenous locus of *Tg*ABCB7L in the ABCE1-HA background, as outlined in Fig. 2A. (N) qRT-PCR analysis of cKD-ABCB7L/ABCE1-HA, as in *B*. (O) Plaque assay of cKD-ABCB7L/ ABCE1-HA as in *C*. (P) Immunofluorescence assay analysis of cKD-ABCB7L/ ABCE1-HA parasites, grown in the presence or absence of ATc for three days, labelled with anti-HA to detect ABCE1-HA (cyan), anti-GAP45 to outline the parasites (magenta). Scale bar is 5 µM. (Q) Immunoblot analysis of whole cell lysate extracted from cKD-ABCB7L/ ABCE1-HA and grown in the presence or absence of ATc for three days. (R) Immunoblot analysis of whole cell lysate extracted from cKD-ABCB7L/ SDHB-HA and grown in the presence or absence of ATc for six days.


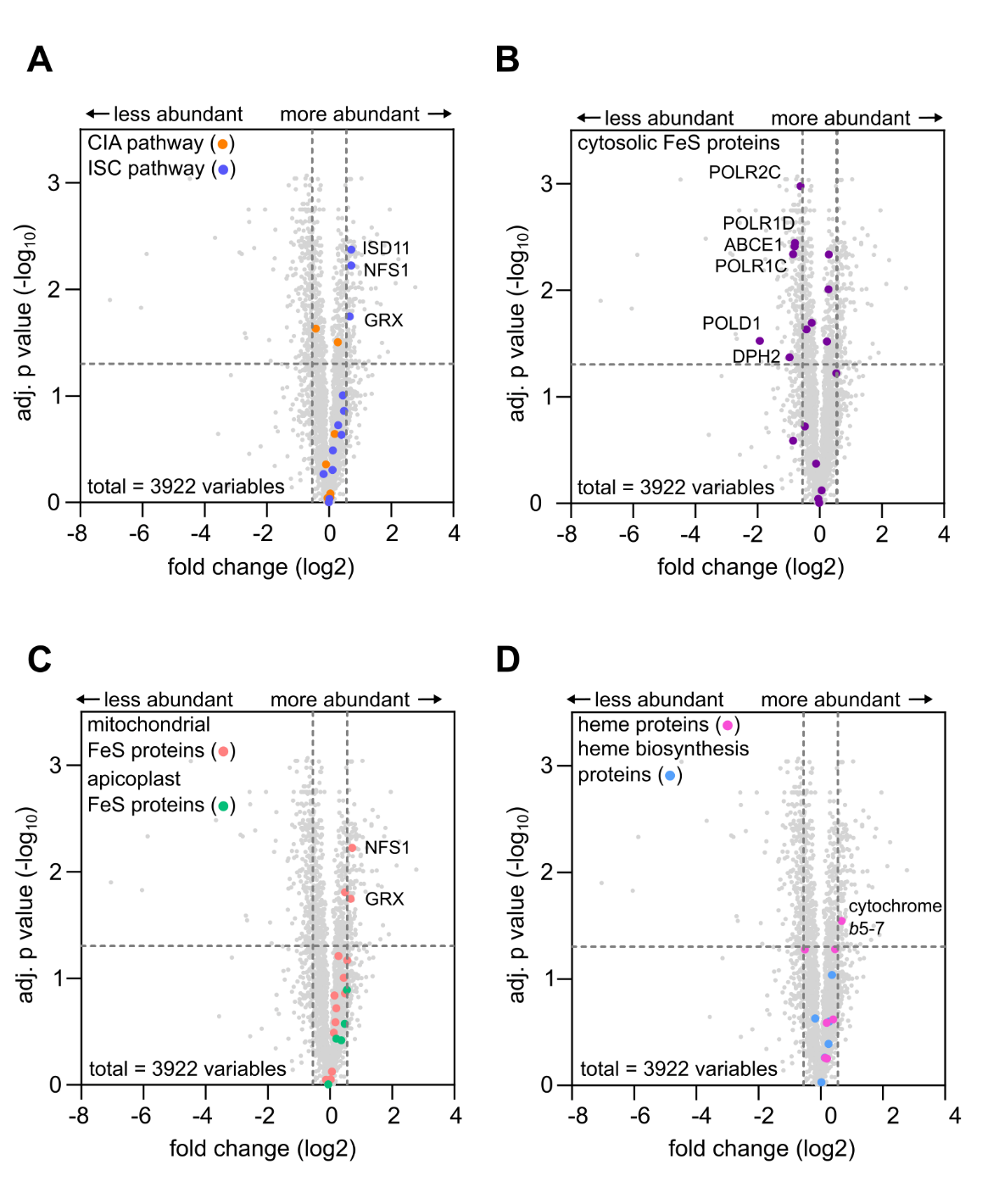


**Figure S6 Volcano plots of CIA and ISC pathway proteins, cytosolic, mitochondrial and apicoplast Fe-S proteins and heme-containing and biosynthetic proteins in the LFQ proteomic dataset.**

Volcano plot showing the difference in protein abundance in cKD-ABCB7L, as in Fig. 5A, for CIA and ISC pathway member (A), for predicted cytosolic (B), mitochondrial and apicoplast localized Fe-S proteins (C) and heme-containing and heme biosynthetic proteins (D) (see Table S1,2). Proteins that are significantly different from the parental control, and above the ± ≥ 0.55 Log2FC cut-off, are individually labelled.

**Supplemental references:**

1. Srinivasan V, Pierik AJ, Lill R. 2014. Crystal structures of nucleotide-free and glutathione-bound mitochondrial ABC transporter Atm1. Science 343:1137–40.

2. Varadi M, Anyango S, Deshpande M, Nair S, Natassia C, Yordanova G, Yuan D, Stroe O, Wood G, Laydon A, Žídek A, Green T, Tunyasuvunakool K, Petersen S, Jumper J, Clancy E, Green R, Vora A, Lutfi M, Figurnov M, Cowie A, Hobbs N, Kohli P, Kleywegt G, Birney E, Hassabis D, Velankar S. 2022. AlphaFold Protein Structure Database: massively expanding the structural coverage of protein-sequence space with high-accuracy models. Nucleic Acids Res 50:D439–D444.

3. Jumper J, Evans R, Pritzel A, Green T, Figurnov M, Ronneberger O, Tunyasuvunakool K, Bates R, Žídek A, Potapenko A, Bridgland A, Meyer C, Kohl SAA, Ballard AJ, Cowie A, Romera-Paredes B, Nikolov S, Jain R, Adler J, Back T, Petersen S, Reiman D, Clancy E, Zielinski M, Steinegger M, Pacholska M, Berghammer T, Bodenstein S, Silver D, Vinyals O, Senior AW, Kavukcuoglu K, Kohli P, Hassabis D. 2021. Highly accurate protein structure prediction with AlphaFold. Nature 596:583–589.

4. Zhang Y, Skolnick J. 2005. TM-align: a protein structure alignment algorithm based on the TM-score. Nucleic Acids Res 33:2302–9.

5. Meng EC, Goddard TD, Pettersen EF, Couch GS, Pearson ZJ, Morris JH, Ferrin TE. 2023. UCSF ChimeraX: Tools for structure building and analysis. Protein Sci 32:e4792.
